# An Fe(III)-covalent organic framework (COF)–sorafenib nanoplatform induces chemo-ferroptosis for enhanced hepatocellular carcinoma immunotherapy

**DOI:** 10.1016/j.mtbio.2025.102135

**Published:** 2025-07-26

**Authors:** Binglong Bai, Zihao Zheng, Bingzi Zhu, Jianlin Wu, Yizhou Xu, Xihao Zhong, Wenhai Deng, Xiang Wang, Shengsheng Zhao, Tao You, Yingpeng Huang, Weijian Sun, Xian Shen, Xufeng Lu

**Affiliations:** a Department of Gastrointestinal Surgery, Zhejiang International Scientific and Technological Cooperation Base of Translational Cancer Research, The Second Affiliated Hospital and Yuying Children's Hospital of Wenzhou Medical University, Wenzhou, Zhejiang, 325000, China; b Department of Hepatobiliary and Pancreatic Surgery, The Second Affiliated Hospital and Yuying Children's Hospital of Wenzhou Medical University, Wenzhou, Zhejiang, 325000, China; c Department of Hepatobiliary and Pancreatic Surgery, The Third Affiliated Hospital of Wenzhou Medical University (Ruian People's Hospital), Wenzhou, Zhejiang, 325000, China; d Zhejiang Key Laboratory of Intelligent Cancer Biomarker Discovery and Translation, Department of Gastrointestinal Surgery, The First Affiliated Hospital, Wenzhou Medical University, Wenzhou, Zhejiang, 325000, China; e Research Center of Basic Medicine, The Second Affiliated Hospital and Yuying Children's Hospital of Wenzhou Medical University, Wenzhou, Zhejiang, 325000, China; f School of Landscape Architecture, Zhejiang Agriculture and Forestry University, Hangzhou, Zhejiang, 311100, China; g Oujiang Laboratory (Zhejiang Lab for Regenerative Medicine, Vision, and Brain Health), School of Laboratory Medicine and Life Sciences, Wenzhou Medical University, Wenzhou, Zhejiang, 325000, China; h Department of Colorectal and Anal Surgery, The First Affiliated Hospital of Wenzhou Medical University, Wenzhou, Zhejiang, 325000, China

**Keywords:** Covalent organic framework (COF), Sorafenib, Chemo-ferroptosis, Hepatocellular carcinoma (HCC), Programmed death receptor-1 (PD-1)

## Abstract

Chemoresistance remains a tremendous challenge in the clinical treatment of hepatocellular carcinoma (HCC). However, the induction of ferroptosis, a form of regulated cell death, could overcome chemoresistance and improve treatment outcomes for HCC patients. In this study, we constructed a covalent organic framework (COF)-based nanoplatform (SRF@Fe(III)-COF) that combines ferroptosis induction and chemotherapy for dual-mode HCC treatment. The peroxidase- and glutathione oxidase-like activities of the nanoplatform are mediated by ferric (Fe(III)) ions, which act as excellent ferroptosis executors, and the encapsulated sorafenib (SRF) can attenuate tumor cell resistance to ferroptosis, effectively facilitating chemo-ferroptosis. Furthermore, SRF@Fe(III)-COF was found to trigger immunogenic cell death and stimulate a pronounced immune response by inducing robust ferroptosis. SRF@Fe(III)-COF synergized with programmed death receptor-1 (PD-1) immune checkpoint blockade to effectively restrict the growth of primary and distal H22 tumors by recruiting tumor-infiltrating lymphocytes and establishing systemic immune memory. Overall, our study revealed that the combination of a chemotherapeutic agent with a ferroptosis inducer is a promising approach for improving HCC immunotherapy.

## Graphical abstract

**Figure:**
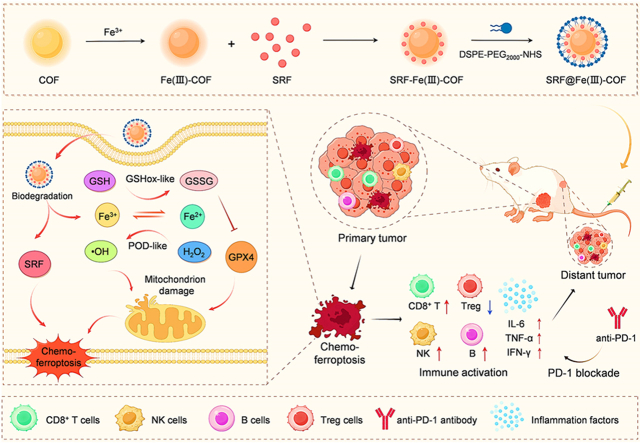

## 1 Introduction

Hepatocellular carcinoma (HCC) is highly aggressive and has limited therapeutic options, especially in advanced stages [1]. The development of systemic therapies for HCC has been challenging because of the complex interplay between the tumor and the underlying liver disease [2]. However, the advent of immunotherapy has led to a paradigm shift in the management of HCC. Immune checkpoint inhibitors (ICIs), which target molecules such as programmed death receptor-1 (PD-1), programmed death ligand 1 (PD-L1), and cytotoxic T-lymphocyte antigen-4 (CTLA-4), have shown promise in modulating the immune response to cancer cells [3]. ICIs enhance the body's natural immune surveillance mechanisms, promoting antitumor activity [4]. Although single-agent ICIs have objective response rates of approximately 15–20 % in HCC patients, their efficacy is often limited by intrinsic resistance mechanisms [1,5]. Therefore, combination therapies are being designed to overcome resistance and broaden the patient population that can benefit from treatment. Combining ICIs with chemotherapeutic drugs or with other ICIs has shown encouraging results in preclinical and early-phase clinical trials [1,6]. Therefore, the integration of immunotherapy into the treatment landscape of HCC has not only improved survival outcomes but also revealed new avenues for research and clinical practice.

Ferroptosis, a classical form of regulated cell death characterized by iron-dependent lipid peroxidation, has attracted considerable attention in the context of cancer biology, particularly for HCC [7,8]. Ferroptosis has been shown to play crucial roles in modulating HCC progression and response to therapy and has been explored as a potential therapeutic approach [9]. Ferroptosis is driven by several key mechanisms, including the regulation of antioxidant defenses, lipid metabolism, and iron homeostasis [10,11]. The efficacy of iron-induced ferroptosis is limited by weak Fenton reactions and high levels of glutathione (GSH) in the tumor microenvironment (TME) [12]. However, sorafenib (SRF), a multikinase inhibitor, has been established as a first-line treatment for advanced HCC [13]. Despite its initial success in prolonging survival and slowing HCC progression, the efficacy of SRF is frequently compromised by the development of resistance in cancer cells, which remains a major clinical challenge [14]. Recent studies have demonstrated the critical role of ferroptosis in modulating the response to SRF. Specifically, SRF has been shown to induce ferroptosis by disrupting the cystine/glutamate antiporter system (system xc-) and depleting GSH levels, leading to increased reactive oxygen species (ROS) levels and thus to lipid peroxidation [15]. However, resistance to SRF can be mediated by pathways that inhibit ferroptosis, such as the activation of antioxidant defenses and the upregulation of ferroptosis-suppressing genes [16]. Ferroptosis has emerged as a critical mechanism that may be altered to increase the sensitivity of HCC to SRF [17]; it is a potential target of combination therapies to overcome resistance and improve outcomes in HCC patients.

**Scheme 1:**
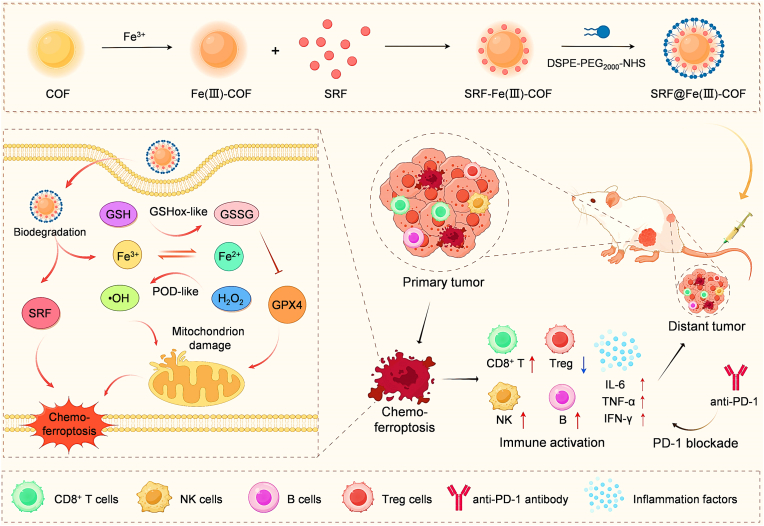
Schematic illustration of the synthesis of SRF@Fe(III)-COF and the mechanism of SRF@Fe(III)-COF-induced chemo-ferroptosis to enhance combined immunotherapy.

Covalent organic frameworks (COFs) represent a burgeoning class of crystalline porous polymers formed through reticular chemistry-mediated reversible covalent bonds [18]. COFs have demonstrated great potential across a variety of applications, including gas storage and separation, catalysis, and drug delivery, owing to their distinctive structural characteristics, such as robust chemical stability, high surface area, and tunable pore sizes [19]. One of the most compelling features of COFs is their potential in the field of nanomedicine, particularly for drug delivery and cancer theranostics [20,21]. Recent studies revealed that iron-induced ferroptosis and SRF-mediated anti-HCC effects have considerable therapeutic efficacy [17]. Because chemoresistance and weak Fenton activity often cause the failure of HCC treatment [17], the combination of ferroptosis inducers with chemotherapy has inherent potential for resolving this problem. Therefore, novel combination therapies to increase ferroptosis sensitivity and overcome SRF resistance are urgently needed to improve treatment outcomes in HCC. In this study, we developed an Fe(III)-COF nanosystem; a self-assembly process enables loading of SRF, and subsequent encapsulation by polyethylene glycol (PEG) enables synergistic dual-mode tumor therapy. The SRF@Fe(III)-COF integrates peroxidase (POD)-like and glutathione oxidase (GSHox)-like activities to facilitate excessive ROS accumulation and decrease the amount of cellular GSH within cancer cells, which decreased the expression of glutathione peroxidase 4 (GPX4) and induced ferroptosis pathways to precisely execute cancer cell, tumor spheroid, and tumor death. Importantly, the increase in ROS accumulation that occurred upon SRF@Fe(III)-COF-induced ferroptosis, in turn, enhanced the anticancer effects of SRF, ultimately resulting in robust cell death. SRF@Fe(III)-COF-mediated chemo-ferroptosis effectively triggered immunogenic cell death (ICD), thereby improving tumor immunogenicity and promoting antitumor responses. Furthermore, in combination with anti-PD-1 therapy, SRF@Fe(III)-COF reversed the inhibitory immune TME, strongly suppressing the growth of primary and distal tumors. These findings suggest that this COF delivery system has the potential to serve as an effective and multifaceted carrier with great promise in HCC immunotherapy (Scheme 1).

## 2 Results

### 2.1 Construction of SRF@Fe(III)-COF

**Fig. 1:**
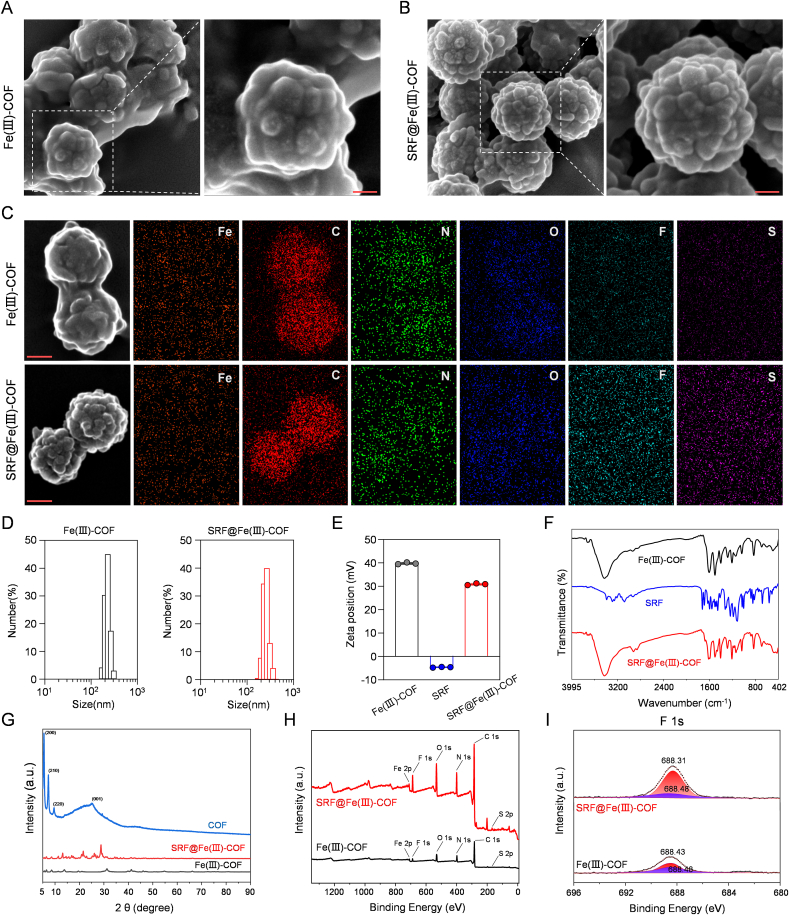
Construction of SRF@Fe(III)-COF. A, B) SEM images of the Fe(III)-COF and SRF@Fe(III)-COF. Scale bar: 50 nm. C) Elemental analysis of the Fe(III)-COF and SRF@Fe(III)-COF. Scale bar: 100 nm. D) Size distribution histogram of the Fe(III)-COF and SRF@Fe(III)-COF. E) Zeta potentials of the Fe(III)-COF, SRF, and SRF@Fe(III)-COF (n = 3). F) FTIR spectra of the Fe(III)-COF, SRF, and SRF@Fe(III)-COF. G) XRD patterns of the COF, Fe(III)-COF and SRF@Fe(III)-COF. H) XPS survey spectrum of the Fe(III)-COF and SRF@Fe(III)-COF I) Binding energy spectrum for F 1s of the Fe(III)-COF and SRF@Fe(III)-COF.The chemical formula of the sorafenib tosylate is C_28_H_24_ClF_3_N_4_O_6_S. Data are presented as mean ± SD.

First, the Fe(III)-COF was synthesized via a two-step method [22] and then conjugated with SRF and encapsulated in PEG to increase its stability and biocompatibility (Scheme 1). Scanning electron microscopy (SEM) and transmission electron microscopy (TEM) revealed that the obtained SRF@Fe(III)-COF exhibited a monodisperse spherical morphology (Fig. 1A–C; Fig. S1, Fig. S2). Additionally, particle size analysis revealed that the diameter of the Fe(III)-COF nanoparticles increased slightly after SRF loading, whereas the zeta potential decreased from 40 to 30 mV (Fig. 1D and E). The successful synthesis of SRF@Fe(III)-COF was confirmed by Fourier transform infrared (FTIR) spectroscopic analyses and ultraviolet–visible (UV–vis) spectrophotometry (Fig. 1F; Fig. S3). The drug loading content (LC%), confirmed by high-performance liquid chromatography (HPLC), was calculated to be 36.01 %. The atomic Fe content of SRF@Fe(III)-COF was measured to be 2.55 % via inductively coupled plasma‒mass spectrometry (ICP‒MS). Furthermore, X-ray diffraction (XRD) analysis of the synthesized SRF@Fe(III)-COF showed a characteristic pattern similar to that of the pristine Fe(III)-COF (Fig. 1G). Elemental analysis and X-ray photoelectron spectroscopy (XPS) revealed the presence of Fe, C, N, and O in the Fe(III)-COF, whereas SRF@Fe(III)-COF contained many more S and F atoms (Fig. 1H and I; Fig. S4). In addition, after SRF loading, the N 1 s and O 1 s binding energy peaks shifted relative to those in the Fe(III)-COF spectrum (Fig. 1H; Fig. S4). The N_2_ adsorption/desorption results revealed that the Brunauer–Emmett–Teller (BET)-specific surface areas of the COF, Fe(III)-COF and SRF@Fe(III)-COF were ∼787.82 m^2^/g, 34.05 m^2^/g and 25.22 m^2^/g, respectively (Fig. S5). These results indicated the successful construction of SRF@Fe(III)-COF.

### 2.2 POD- and GSHox-like activities of SRF@Fe(III)-COF

**Fig. 2:**
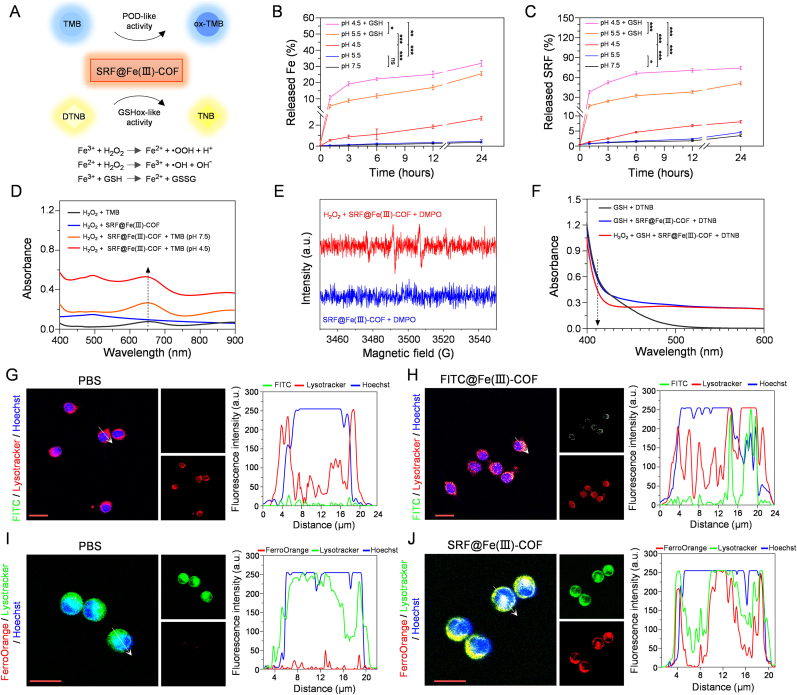
Nanozyme activity and cellular uptake of SRF@Fe(III)-COF. A) Schematic illustration of POD-like and GSHox-like activities of SRF@Fe(III)-COF. B, C) Cumulative release of Fe and SRF from SRF@Fe(III)-COF in NaAc solution with various pH and GSH concentrations (n = 3). D) Determination of POD-like activity of SRF@Fe(III)-COF using TMB as a substrate. E) Detection of •OH generation was performed using EPR spectroscopy, with DMPO serving as the spin trap for •OH. F) Determination of GSHox-like activity of SRF@Fe(III)-COF using DTNB as a substrate. G, H) Subcellular colocalization images of FITC modified Fe(III)-COF (green) in H22 cells. Nucleus was labeled with Hoechst (blue), and lysosomes were visualized using Lysotracker (red). Colocalization fluorescence intensity in H22 cells (white arrow) was quantified and depicted in line plots. Scale bar: 25 μm. I, J) Fluorescence images of intracellular Fe levels (red) in H22 cells. Nucleus was labeled with Hoechst (blue), and lysosomes were visualized using Lysotracker (green). Colocalization fluorescence intensity in H22 cells (white arrow) was quantified and depicted in line plots. Scale bar: 20 μm. Data are presented as mean ± SD; ∗p < 0.05; ∗∗p < 0.01; ∗∗∗p < 0.001; ns, not significant.

Fe(III)-loaded COF nanoparticles exhibit superior nanozyme activity upon the conversion of Fe(III) to Fe(II) [23]. First, the concentration of Fe released from the material was measured via ICP‒MS. SRF@Fe(III)-COF released 0.37 % of the Fe after incubation in neutral NaAc buffer (pH 7.5) for 24 h (Fig. 2A and B). In contrast, SRF@Fe(III)-COF exhibited significant pH-responsive biodegradation at pH 5.5 and 4.5 and released 0.51 % and 2.64 % of the Fe after 24 h, respectively (Fig. 2B), demonstrating the relative stability of SRF@Fe(III)-COF under physiological conditions. In addition, SRF was shown by HPLC to be released from SRF@Fe(III)-COF under acidic conditions (Fig. 2C), suggesting that SRF release occurs in a pH-sensitive manner. Since the intracellular environment or TME contains high GSH levels [12], we further investigated the biodegradation behavior of SRF@Fe(III)-COF at various pH values and GSH concentrations. Notably, approximately 30 % of the Fe and 70 % of the SRF were released from SRF@Fe(III)-COF at pH 4.5 and 10 mM GSH (Fig. 2B and C), further confirming that PEG-encapsulated SRF@Fe(III)-COF NPs improved their stability in acidic environments. In conclusion, SRF@Fe(III)-COF has good biodegradability inside tumor cells, enhancing therapeutic efficacy while minimizing systemic toxicity.

The POD-like activity of SRF@Fe(III)-COF was evaluated colorimetrically upon 3,3′,5,5′-tetramethylbenzidine (TMB) oxidation via a Fenton-like reaction (Fig. 2A). As shown in Fig. 2D, SRF@Fe(III)-COF exhibited weak POD-like activity at neutral pH, whereas this activity was significantly enhanced under acidic conditions. This pH-dependent activity suggests that SRF@Fe(III)-COF can selectively target the TME. To further investigate the type of hydroxyl radical (•OH) generated, an electron paramagnetic resonance (EPR) assay was conducted using 5,5-dimethyl-1-pyrroline N-oxide (DMPO) [24]. As depicted in Fig. 2E, compared with the control sample, the SRF@Fe(III)-COF sample presented a more intense DMPO-OH signal, confirming its excellent POD-like activity.

The extremely high levels of GSH in the TME can efficiently neutralize the ROS generated by SRF@Fe(III)-COF, thereby eliminating its catalytic therapeutic efficacy [25]. Given that Fe(III) can catalyze the oxidation of the GSH thiol group [26], we explored the GSHox-like activity of SRF@Fe(III)-COF using the probe 5,5′-dithiobis(2-nitrobenzoic acid) (DTNB) (Fig. 2A). DTNB reacts with residual sulfhydryl groups on GSH to form a yellow compound (the 2-nitro-5-thiobenzoic acid anion, TNB) [27]. As shown in Fig. 2F, SRF@Fe(III)-COF demonstrated a robust capacity to deplete GSH. The GSHox-like activity of SRF@Fe(III)-COF was further assessed with a GSH tracer, which revealed a shift in the fluorescence excitation and emission peaks from Ex/Em = 520/580 nm to Ex/Em = 430/510 nm upon binding with GSH [28]. Similarly, the fluorescence ratio of Em = 510 nm to Em = 580 nm significantly decreased after GSH was treated with SRF@Fe(III)-COF and H_2_O_2_ (Fig. S6). Collectively, these findings suggest that SRF@Fe(III)-COF can effectively deplete GSH through its GSHox-like activity.

### 2.3 Cellular uptake

Encouraged by the outstanding catalytic properties of SRF@Fe(III)-COF (Fig. 2A–F; Fig. S6), we conducted *in vitro* studies to assess its cytotoxic potential. Initially, the cellular uptake of fluorescein isothiocyanate (FITC)-labeled Fe(III)-COF was examined using confocal laser scanning microscopy (CLSM). As shown in Fig. 2G and H, a substantial increase in green fluorescence was observed in H22 cells after 8 h of incubation. The colocalization of a lysosome tracker and FITC within tumor cells was subsequently evaluated to investigate the subcellular localization of the nanoplatform. CLSM revealed that FITC@Fe(III)-COF effectively entered the lysosomes, facilitating its catalytic activity. Flow cytometry analysis indicated that the fluorescence signal in the FITC@Fe(III)-COF-treated H22 cells was significantly greater than that in the control cells (Fig. S7). Considering the critical role of the intracellular Fe concentration in ferroptosis [29], we employed ICP‒MS to quantify Fe accumulation in H22 cells. The intracellular Fe content in the cells treated with the FITC@Fe(III)-COF for 8 h was significantly greater than that in the control cells (Fig. S8). Additionally, the fluorescence intensity of Fe in SRF@Fe(III)-COF-treated H22 cells was dramatically greater than that in phosphate-buffered saline (PBS)-treated cells, as determined using the FerroOrange probe (Fig. 2I and J). Collectively, these results demonstrate that SRF@Fe(III)-COF was rapidly internalized by H22 cells and exhibited antitumor activity.

### 2.4 Cytotoxicity of SRF@Fe(III)-COF

**Fig. 3:**
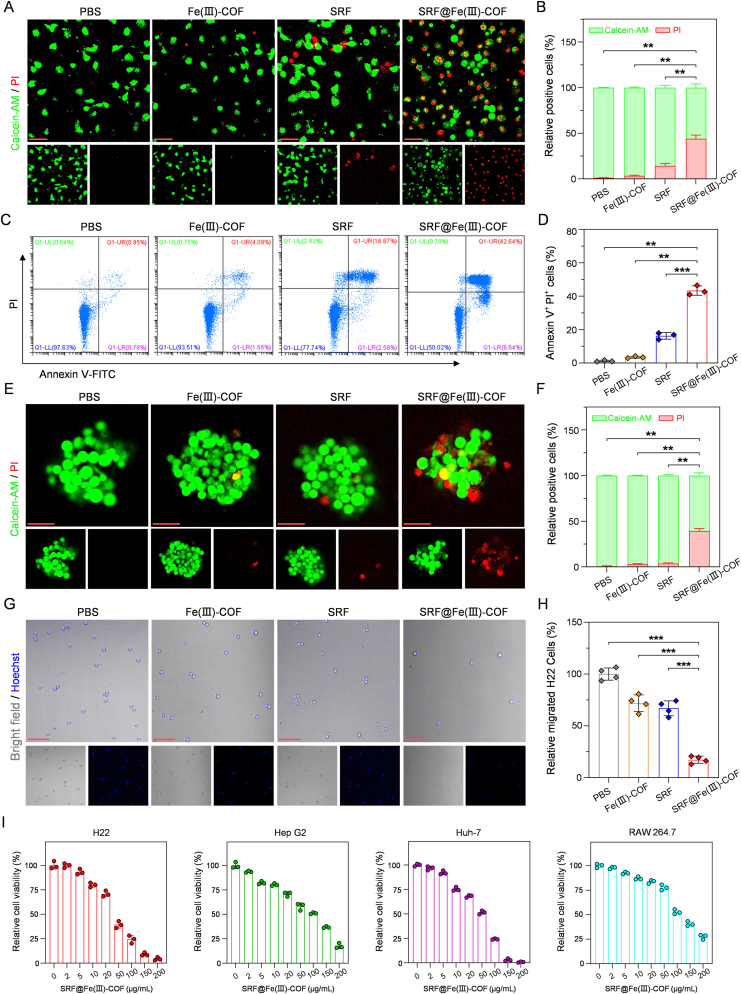
Cytotoxicity of SRF@Fe(III)-COF *in vitro*. A) Live/dead fluorescent images of H22 cells stained by calceinAM and PI, respectively. Scale bar: 50 μm. B) Quantitative analysis of calcein-AM-positive and PI-positive H22 cells following 24 h of exposure to various treatments (n = 3). C) Flow cytometric assays of the Annexin V-FITC/PI-stained H22 cells after different treatments. D) Quantitative analysis of Annexin V and PI double-positive H22 cells following various treatments (n = 3). E) Images and cell viability of 3-dimensional (3D) H22 culture spheroids following various treatments. Scale bar: 50 μm. F) Quantitative analysis of calcein-AM-positive and PI-positive cells in 3D H22 culture spheroids after various treatments (n = 3). G, H) The migration of suspension H22 cells with various treatments (n = 4). Nucleus was labeled with Hoechst (blue). Scale bar: 50 μm. I) Relative cell viability of H22, HepG2, Huh-7 and RAW264.7 cells after 24 h of incubation with different concentrations of SRF@Fe(III)-COF (n = 3). Data are presented as mean ± SD; ∗∗p < 0.01; ∗∗∗p < 0.001.

Next, calcein-AM and propidium iodide (PI) staining were performed to confirm the antitumor effects of SRF@Fe(III)-COF in H22 cells. As depicted in Fig. 3A and B, treatment with Fe(III)-COF or SRF alone had little effect on H22 cells. However, treatment with SRF@Fe(III)-COF resulted in a high growth inhibition rate (Fig. 3A and B), indicating that combining Fe(III)-COF with SRF led to anticancer effects that were superior to those of either material alone. Furthermore, flow cytometry analysis confirmed the presence of many apoptotic and necrotic cells in the SRF@Fe(III)-COF-treated group (Fig. 3C and D) and thus the remarkable antitumor efficacy of SRF@Fe(III)-COF. To test the hypothesis that SRF@Fe(III)-COF-induced ferroptosis prevents tumor growth, a sphere formation assay was conducted [30]. As expected, SRF@Fe(III)-COF markedly impeded the formation of H22 tumor cell spheres, as determined by live/dead staining experiments (Fig. 3E and F). Moreover, SRF@Fe(III)-COF effectively inhibited the migration of H22 cells (Fig. 3G and H). The antitumor efficacy of SRF@Fe(III)-COF was subsequently evaluated using a Cell Counting Kit-8 (CCK-8) assay. At 50 μg/mL, the growth inhibition rate of the H22 cells in the SRF@Fe(III)-COF group exceeded 50 % (Fig. 3I; Fig. S9). Furthermore, SRF@Fe(III)-COF exhibited similar cytotoxic effects on human HCC cell lines (HepG2 and Huh-7) and mouse macrophages (RAW 264.7) (Fig. 3I). In conclusion, these results demonstrated that SRF@Fe(III)-COF has significant anti-HCC effects.

### 2.5 SRF@Fe(III)-COF triggered chemo-ferroptosis

**Fig. 4:**
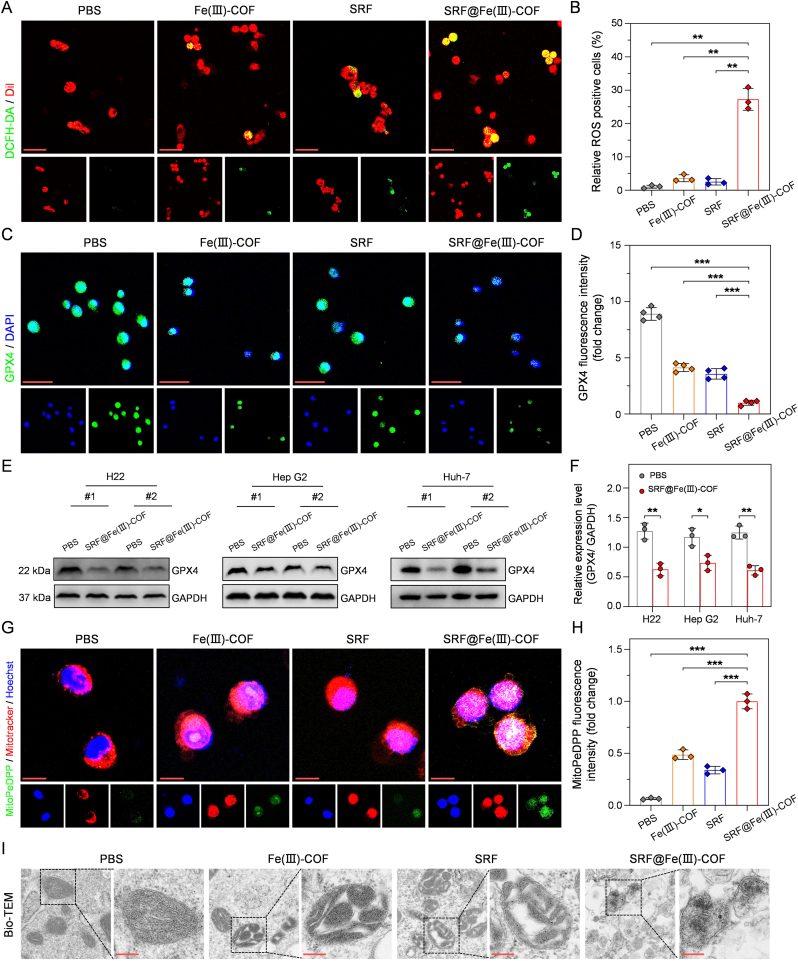
SRF@Fe(III)-COF triggered chemo-ferroptosis *in vitro*. A) CLSM images of ROS levels (green) in Dil-stained H22 cells following various treatments. Scale bar: 50 μm. B) Quantitative analysis of ROS levels in H22 cells after the various treatments (n = 3). C) CLSM images of GPX4 (green) in the H22 cells following various treatments. Scale bar: 50 μm. The nucleus was labeled with DAPI (blue). D) Quantitative analysis of GPX4 fluorescence intensity in H22 cells after the indicated treatments (n = 4).E, F) Protein expression of GPX4 in H22, HepG2, and Huh-7 cells after SRF@Fe(III)-COF treatment, as evaluated by western blot analysis. GAPDH was used as a loading control. The intensity of the blot was quantified using ImageJ software.G) Mitochondrial lipid peroxidation was evaluated by MitoPeDPP probe (green). Nucleus was labeled with Hoechst (blue), and mitochondria were visualized using Mitotracker (red). Scale bar: 10 μm. H) Quantitative analysis of MitoPeDPP fluorescence intensity in H22 cells after the indicated treatments (n = 3). I) Representative bio-TEM images of mitochondria shrinkage after indicated treatments. Scale bar: 200 nm. Data are presented as mean ± SD; ∗p < 0.05; ∗∗p < 0.01; ∗∗∗p < 0.001.

To elucidate the potential mechanism of the antitumor effect of SRF@Fe(III)-COF, we explored the pathways involved in tumor cell death. The ROS-sensitive probe 2,7-dichlorodihydrofluorescein diacetate (DCFH-DA) was used to assess the cellular ROS levels after the indicated treatments [31]. As shown in Fig. 4A and B, H22 cells treated with SRF presented minimal fluorescence signals, further confirming that SRF is a weak inducer of ferroptosis in tumor cells [14]. Compared with cells treated with Fe(III)-COF alone, those treated with SRF@Fe(III)-COF displayed significantly higher green fluorescence signals (Fig. 4A and B), indicating that resistance to SRF using the Fenton reaction was overcome in these cancer cells. However, the generated ROS can be rapidly neutralized by high levels of GSH in tumor cells [25]. Given the GSHox-like capacity of SRF@Fe(III)-COF (Fig. 2A and F; Fig. S6), the GSH levels in H22 cells treated with SRF@Fe(III)-COF were substantially reduced. These results demonstrated that SRF@Fe(III)-COF effectively depleted GSH owing to its GSHox-like activity (Fig. S10), thereby increasing oxidative stress and inducing ferroptosis in cells. GSH depletion can lead to GPX4 inactivation and increased oxidative stress, ultimately resulting in ferroptosis. As expected, the green fluorescence intensity of GPX4 in H22 cells significantly decreased upon treatment with SRF@Fe(III)-COF (Fig. 4C and D), which was attributed to its ability to deplete GSH and further confirmed its GSHox-like activity. Furthermore, western blot analysis revealed that Fe(III)-COF and SRF@Fe(III)-COF effectively downregulated GPX4 in H22 cells and human HCC cell lines (HepG2 and Huh-7) (Fig. 4E and F; Fig. S11), confirming its ability to induce ferroptosis in HCC cells. Next, we examined the oxidation of mitochondrial membrane lipids using the MitoPeDPP probe, which is a mitochondria-specific lipid peroxidation imaging dye [32]. An increase in the intensity of MitoPeDPP was observed in H22 cells after SRF@Fe(III)-COF treatment (Fig. 4G and H), suggesting that the oxidation of mitochondrial membrane lipids was significantly induced by SRF@Fe(III)-COF. We further analyzed the morphology of the cellular mitochondria via biological transmission electron microscopy (Bio-TEM) [33]. Compared with control H22 cells, H22 cells treated with SRF@Fe(III)-COF exhibited noticeable mitochondrial shrinkage (Fig. 4I), a hallmark of ferroptosis [33]. Collectively, the above results indicated that the catalytic activity of Fe and the cytotoxicity of SRF contributed synergistically to the induction of chemo-ferroptosis.

### 2.6 SRF@Fe(III)-COF-triggered ICD

**Fig. 5:**
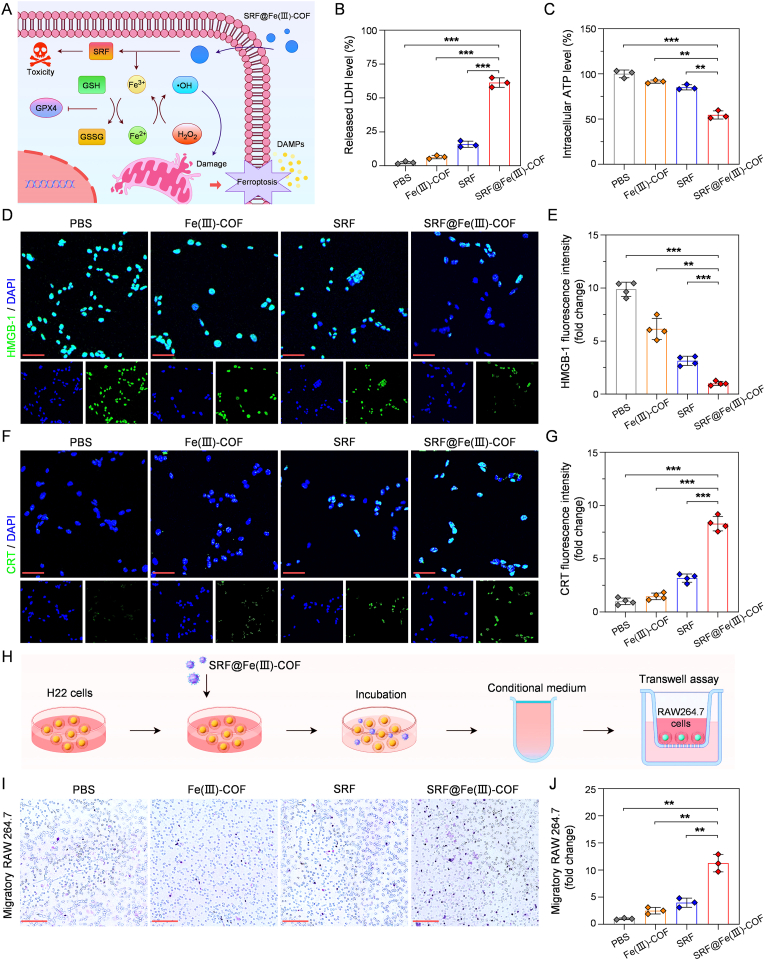
SRF@Fe(III)-COF triggered ICD *in vitro*. A) Schematic illustration of the mechanism underlying ICD induced by SRF@Fe(III)-COF. B) Levels of LDH released into the extracellular matrix following the various treatments (n = 3). C) The relative levels of intracellular ATP in H22 cells after various treatments (n = 3). D) CLSM images of HMGB-1 (green) in the H22 cells following various treatments. Scale bar: 50 μm. The nucleus was labeled with DAPI (blue). E) Quantitative analysis of HMGB-1 fluorescence intensity in H22 cells after the indicated treatments (n = 4). F) CLSM images of CRT (green) in the H22 cells following various treatments. Scale bar: 50 μm. The nucleus was labeled with DAPI (blue). G) Quantitative analysis of CRT fluorescence intensity in H22 cells after various treatments (n = 4). H) Schematic illustration of the transwell co-culture system simulating chemotaxis of RAW264.7 cells to conditional medium. I) Representative images of RAW 264.7 cells cultured on transwell stimulated with the indicated conditional media. Scale bars: 200 μm. J) Quantitative analysis of chemotaxis RAW264.7 cells after indicated treatments (n = 3). Data are presented as mean ± SD; ∗∗p < 0.01; ∗∗∗p < 0.001.

To evaluate the SRF@Fe(III)-COF-triggered antitumor immune response, we analyzed several damage-associated molecular patterns (DAMPs) *in vitro* (Fig. 5A). Compared with those in the other groups, significantly more lactate dehydrogenase (LDH) and adenosine triphosphate (ATP) were released into the extracellular environment following treatment with SRF@Fe(III)-COF (Fig. 5B and C), suggesting that SRF@Fe(III)-COF-mediated ferroptosis significantly promoted the release of DAMPs into the TME. Additionally, the effects of exposure to high-mobility group box-1 (HMGB-1) and calreticulin (CRT) after various treatments were further analyzed. Among all the treatments, the SRF@Fe(III)-COF treatment led to substantial HMGB-1 release and the greatest CRT exposure (Fig. 5D–G). DAMPs serve as key signals to induce a primary immune response [34]. The recruitment of antigen-presenting cells was further analyzed using a Transwell assay (Fig. 5H). Conditioned medium from H22 cells treated with SRF@Fe(III)-COF induced significant migration of RAW264.7 cells (Fig. 5I and J). These results indicate that SRF@Fe(III)-COF significantly increased the immunogenicity of H22 cells by triggering ICD. Collectively, these findings demonstrated that ferroptosis induced by SRF@Fe(III)-COF enhanced the antitumor immune response.

### 2.7 Biodistribution

**Fig. 6:**
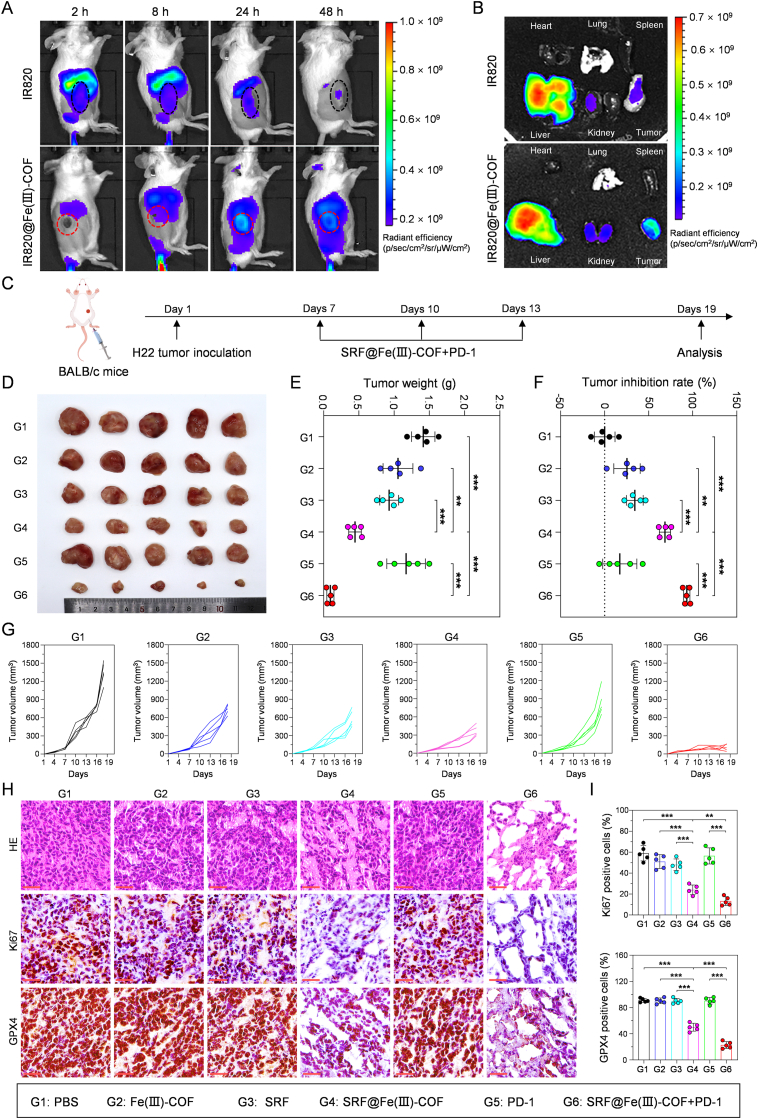
Combining SRF@Fe(III)-COF with PD-1 therapy prevented H22 tumor growth. A) IVIS images of H22 tumor-bearing mice at different time points after intravenous injection B) *Ex vivo* IVIS images of the tumors and major organs collected 48 h post-injection. C) The treatment schedule for the *in vivo* antitumor efficacy of the SRF@Fe(III)-COF + PD-1 combination therapy. D-G) Tumor images (D), tumor weights (E), tumor inhibition rate (F), and tumor growth curves (G) in H22 tumor-bearing mice with different treatments (n = 5). H) H&E, Ki-67, and GPX4 staining of tumor tissues after indicated treatments. Scale bars: 30 μm. I) Quantitative analysis of Ki-67-positive (nuclear positive) and GPX4-positive (cytoplasmic positive) cells in tumor sections (n = 5). Data are presented as mean ± SD; ∗∗p < 0.01; ∗∗∗p < 0.001.

Before the mice were treated, indocyanine green (IR820)-loaded Fe(III)-COF (IR820@Fe(III)-COF) was utilized to monitor the biodistribution of the nanoplatform. The fluorescence signal in the tumor area obviously accumulated over 2 h and peaked 24 h after IR820@Fe(III)-COF injection. Moreover, the fluorescence intensity remained robust for up to 48 h after IR820@Fe(III)-COF was administered (Fig. 6A; Fig. S12A). The tumors, hearts, lungs, livers, kidneys, and spleens were subsequently harvested for *ex vivo* biodistribution analysis. As expected, the fluorescence signal in the excised tumors was markedly brighter than that in the control group of mice at 48 h post injection (Fig. 6B; Fig. S12B). Collectively, these findings demonstrated the sustained retention and effective tumor-targeting ability of SRF@Fe(III)-COF.

### 2.8 Combining SRF@Fe(III)-COF with PD-1 for antitumor therapy

PD-1 is a critical immune checkpoint molecule expressed on the surface of T cells, and the binding of its ligands PD-L1/L2 suppresses T-cell proliferation and cytokine production, ultimately resulting in immune evasion by cancer cells [35]. In HCC, the upregulation of PD-1 and PD-L1 contributes to the suppression of antitumor immunity, but the development of PD-1 inhibitors has revolutionized the treatment of advanced HCC [36]. Given the potent antitumor effects and induction of ICD by SRF@Fe(III)-COF, its therapeutic efficacy was evaluated in H22 tumor-bearing mice. The mice were randomly assigned to six groups: the PBS, Fe(III)-COF, SRF, SRF@Fe(III)-COF, PD-1, and SRF@Fe(III)-COF + PD-1 groups (Fig. 6C). Compared with PBS treatment, SRF@Fe(III)-COF + PD-1 significantly inhibited the increase in tumor weight (Fig. 6D–F). As expected, the SRF@Fe(III)-COF group showed a modest reduction in tumor growth compared with the groups treated with SRF, Fe(III)-COF, or PD-1 alone (Fig. 6D–F). These changes in tumor weight correlated with the visual observations and tumor volume measurements across all groups (Fig. 6D–G; Fig. S13). Notably, the combination of SRF@Fe(III)-COF and an anti-PD-1 antibody achieved the highest antitumor efficacy, with a tumor inhibition rate exceeding 93 %, which surpassed the rates observed with SRF@Fe(III)-COF (68 %), SRF (34 %), and anti-PD-1 (17 %) alone (Fig. 6F). These results highlight the remarkable antitumor potential of combination therapy.

**Fig. 7:**
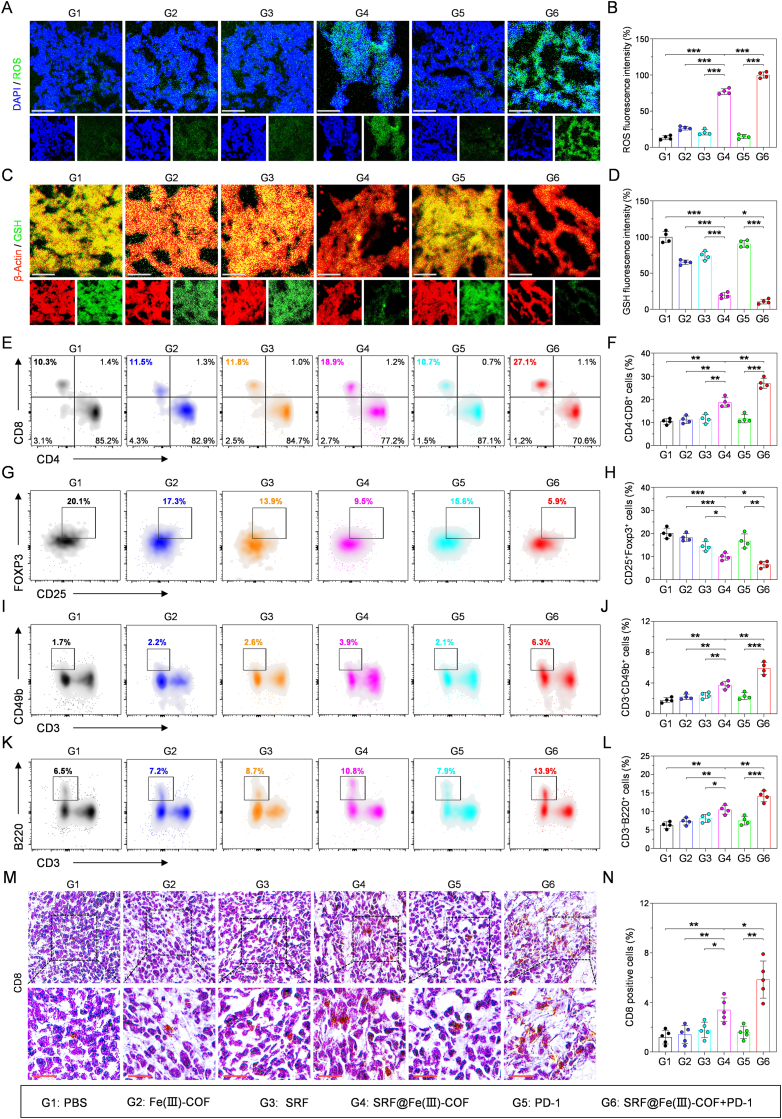
Combining SRF@Fe(III)-COF with PD-1 therapy activated the antitumor immune response. A-D) Immunofluorescence images and quantitative analysis of tumor tissues collected from mice with various treatments and stained with DCFH-DA (ROS-sensitive probe) and ThioTrackerviolet dyes (GSH-sensitive probe) (n = 4). Scale bars: 50 μm. E, F) Flow cytometry and quantitative analysis of CD8^+^ CTLs in tumor tissues following diverse treatments (n = 4). G, H) Flow cytometry and quantitative analysis of CD25^+^FOXP3^+^ Tregs in tumor tissues following diverse treatments (n = 4). I, J) Flow cytometry and quantitative analysis of CD3^-^CD49b^+^ NK cells in tumor tissues following diverse treatments (n = 4). K, L) Flow cytometry and quantitative analysis of CD3^-^B220^+^ lymphocytes in tumor tissues following diverse treatments (n = 4). M, N) Representative IHC images and quantitative analysis of CD8^+^ CTLs in tumor tissues following diverse treatments (n = 5). Scale bars: 20 μm. Data are presented as mean ± SD; ∗p < 0.05; ∗∗p < 0.01; ∗∗∗p < 0.001.

Histopathological analysis further confirmed the significant antitumor efficacy of combined treatment with SRF@Fe(III)-COF and an anti-PD-1 antibody. Hematoxylin and eosin (H&E) staining revealed extensive tissue vacuolation, significant necrosis, and nuclear fragmentation in the SRF@Fe(III)-COF + PD-1 group (Fig. 6H). Moreover, Ki67 staining significantly suppressed tumor proliferation (Fig. 6H and I), further confirming the antitumor efficacy of the combined treatment. Immunohistochemical staining of GPX4 revealed that the SRF@Fe(III)-COF + PD-1 group had a negligible positive index compared with that of the control group (Fig. 6H and I), confirming that SRF@Fe(III)-COF-induced ferroptosis effectively decreased GPX4 expression. Considering the POD- and GSHox-like activities of SRF@Fe(III)-COF, the ROS and GSH levels in tumor tissues were investigated by DCFH-DA and ThiolTracker staining. As expected, high levels of ROS and decreased levels of GSH were observed in the SRF@Fe(III)-COF and SRF@Fe(III)-COF + PD-1 groups compared with those in the PBS and PD-1 groups (Fig. 7A–D), demonstrating the cooperative effect of the production of ROS and depletion of GSH in the SRF@Fe(III)-COF group. These findings demonstrated that ferroptosis triggered by SRF@Fe(III)-COF significantly increased the efficacy of PD-1-targeted therapy for HCC.

### 2.9 Combining SRF@Fe(III)-COF with PD-1 therapy remodeled the immunosuppressive TME

We subsequently investigated how SRF@Fe(III)-COF + PD-1 combination therapy reshaped the immunosuppressive TME (Fig. S14). CD8^+^ T lymphocytes, which are the primary cytotoxic T lymphocytes (CTLs) responsible for the direct elimination of tumor cells [37], were more abundant in the tumors of the mice treated with SRF@Fe(III)-COF + PD-1 than in those treated with SRF@Fe(III)-COF or PD-1 alone (Fig. 7E and F). Moreover, the proportion of regulatory T cells (Tregs), which are crucial for immune evasion [38], was obviously decreased in the tumors of the mice following combined treatment with SRF@Fe(III)-COF + PD-1 (Fig. 7G and H). The tumor-killing capacity of infiltrating natural killer (NK) cells [38] in tumors was subsequently examined, and the tumors of the mice treated with SRF@Fe(III)-COF + PD-1 combination therapy had a greater density of NK cells than those in the other treatment groups did (Fig. 7I and J). Additionally, the ratio of B cells, which are essential for antibody production and antigen recognition [39], was notably elevated in the tumors of the mice treated with SRF@Fe(III)-COF + PD-1 (Fig. 7K and L). Notably, the immunohistochemistry (IHC) results revealed a significant increase in the number of CD8-positive T cells in the tumor tissue after treatment with SRF@Fe(III)-COF + PD-1 (Fig. 7M and N), indicating increased T-cell activation.

### 2.10 Combination therapy suppressed distant tumor growth

**Fig. 8:**
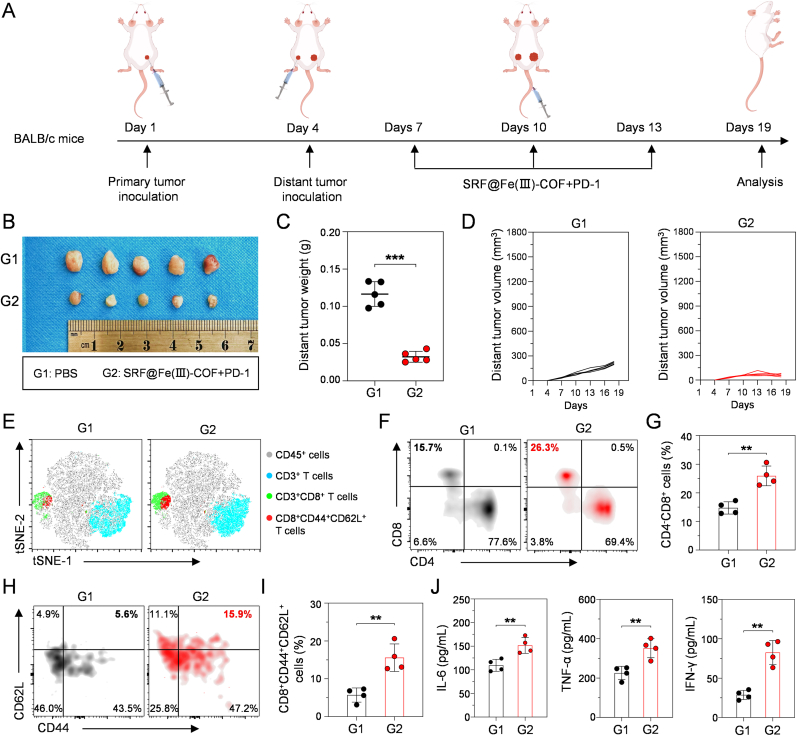
Combining SRF@Fe(III)-COF with PD-1 therapy suppressed distant tumor growth. A) Schematic illustration of distal tumor inoculation and therapeutic regimen. B-D) Images (B), weights (C), and growth curves (D) of distant tumor following indicated treatments (n = 5). E) t-SNE analysis of the distribution of CD8^+^ CTLs and central memory T cells in tumor-infiltrating lymphocytes. F, G) Flow cytometry and quantitative analysis of CD8^+^ CTLs in distant tumor tissues after indicated treatments (n = 4). H, I) Flow cytometry and quantitative analysis of central memory T cells in distant tumor tissues following indicated treatments (n = 4). J) Cytokine levels of IFN-γ, TNF-α, and IL-6 in blood serum after various treatments (n = 4). Data are presented as mean ± SD; ∗∗p < 0.01; ∗∗∗p < 0.001.

To evaluate the therapeutic impact of combination therapy on distant tumors, a distal tumor mouse model was established [40]. H22 cells were injected subcutaneously into the right flank of each mouse to form the primary tumor, and a second (distant) tumor was established by inoculation into the left flank on day 4 (Fig. 8A). The tumor-bearing mice were subsequently treated with PBS or SRF@Fe(III)-COF + PD-1. As expected, the distant tumors grew rapidly in the PBS group. In contrast, mice treated with SRF@Fe(III)-COF + PD-1 presented a 73 % distant tumor growth inhibition rate (Fig. 8B–D; Fig. S15-S16), indicating the abscopal effect of combination therapy. To further explore the robust efficacy against distant tumors, the memory immune responses in the tumors were analyzed (Fig. S14). A t-distributed stochastic neighborhood embedding (t-SNE) analysis revealed that central memory T cells (CD8^+^CD44^+^CD62L^+^), which provide long-lasting immune protection and a rapid immune response against tumors [41], were clearly increased in the distant tumors of the mice after combination therapy (Fig. 8E). Additionally, flow cytometric analysis revealed that, compared with control treatment, SRF@Fe(III)-COF + PD-1 treatment increased the proportion of central memory T cells (Fig. 8F–I). To further assess memory immune response activation, the levels of inflammatory cytokines in mouse blood serum were determined using enzyme-linked immunosorbent assays (ELISAs). The SRF@Fe(III)-COF + PD-1 treatment group presented significant increases in interleukin 6 (IL-6), tumor necrosis factor-α (TNF-α), and interferon-γ (IFN-γ) levels (Fig. 8J), which is consistent with the roles of these molecules in immune responses. Our findings suggested that combination therapy effectively activated long-term antitumor responses and significantly inhibited distant tumor growth.

### 2.11 Biosafety

Additionally, the safety and biocompatibility of SRF@Fe(III)-COF + PD-1 combined therapy were assessed in mice by monitoring the levels of blood biochemical markers, including blood urea nitrogen (BUN), alanine transaminase (ALT), and aspartate aminotransferase (AST) [42], following intravenous injection. No significant changes in these biochemical markers were observed compared with those in the control group (Fig. S17). Moreover, no substantial alterations in body weight were noted during the therapy period (Fig. S18), further supporting the favorable biosafety profile of SRF@Fe(III)-COF + PD-1 combined therapy. As expected, H&E staining revealed no discernible damage to the major organs of the mice treated with SRF@Fe(III)-COF + PD-1 (Fig. S19). A red blood cell hemolysis assay indicated that SRF@Fe(III)-COF caused minimal hemolysis, even at a high concentration of 100 μg/mL (Fig. S20), confirming the biocompatibility of this nanoplatform. In addition, the colloidal stability of SRF@Fe(III)-COF could facilitate its biological application. Therefore, we tested the colloidal stability of SRF@Fe(III)-COF in ultrapure water, PBS, and Roswell Park Memorial Institute 1640 medium (RPMI-1640). As shown in Fig. S21, SRF@Fe(III)-COF exhibited good colloidal stability under physiological conditions. Collectively, these findings confirmed the excellent biosafety of the SRF@Fe(III)-COF + PD-1 combination therapy.

## 3 Conclusion

In summary, we constructed a novel cytotoxic nanoplatform (SRF@Fe(III)-COF) with catalytic activity to induce ferroptosis for HCC therapy. SRF@Fe(III)-COF triggers ferroptosis through a dual mechanism. First, the excellent POD- and GSHox-like activities enable the SRF@Fe(III)-COF to efficiently generate highly toxic ROS and consume cytosolic GSH, inducing GPX4 depletion and cellular oxidative stress and thus resulting in ferroptosis. Second, the released SRF sensitizes cancer cells to ferroptosis and further promoted ferroptotic cell death. The *in vivo* results confirmed that SRF@Fe(III)-COF not only significantly suppressed tumor progression but also increased the efficacy of anti-PD-1 therapy via the ferroptosis pathway, with negligible side effects. Owing to its ICD properties, the SRF@Fe(III)-COF reshaped the TME by stimulating an antitumor immune response that synergized with PD-1‒PD-L1/L2 signaling blockade within the TME. Therefore, primary and distant tumor growth were inhibited *in vivo*. In recent studies, various metal-based nanomaterials have been extensively developed for tumor immunotherapy [43,44]. However, achieving favorable treatment outcomes in HCC patients is difficult because of the relatively low rate of the Fenton reaction, resistance to SRF, and the immunosuppressive TME [17]. Our findings demonstrated that, compared with ferroptosis-based monotherapy or chemotherapy, SRF@Fe(III)-COF successfully integrated the induction of ferroptosis with chemotherapeutic effects in a temporally coordinated manner (Scheme 1). This effective chemo-ferroptosis based antitumor strategy, characterized by decreased side effects and increased efficacy, holds promise for combination immunotherapy.

## 4 Experimental section

### 4.1 Synthesis of Fe(III)-COF

COF was synthesized according to previous method [45]. 20 mg of 2,5-dimethoxybenzene-1,4-dicarboxaldehyde (DMTP), 25 mg of 1,3,5-tris(4-aminophenyl)benzene (TAPB), 50 mL of acetonitrile, and 1.25 mL of acetic acid were mixed to stir at room temperature for 24 h. Subsequently, 25 mg of synthesized COF and 125 mg of FeCl_3_ were dissolved in 50 mL of ddH_2_O, and the mixture was stirred for 24 h. After that, 50 mL of a p-phenylenediamine solution (5 mg/mL) was added into the reaction, and then the mixture was stirred for 24 h [22]. Fe(III)-COF nanoparticles were obtained via centrifugation and washed three times with ddH_2_O.

### 4.2 Synthesis of SRF@Fe(III)-COF

10 mg of Fe(III)-COF, 10 mg of DSPE-PEG_2000_-NHS, and 10 mL of SRF solution (2 mg/mL) were mixed and stirred for 24 h. The obtained SRF@Fe(III)-COF was subsequently washed three times with ultrapure water. The synthesis of FITC@Fe(III)-COF and IR820@Fe(III)-COF followed a similar procedure.

### 4.3 SRF loading efficiency

The calibration curve for SRF was determined using the HPLC method [46]. The encapsulation efficiency of SRF was assessed by quantifying the residual SRF following centrifugation (12,000×*g*, 15 min). The drug-loading content (LC%) of SRF was determined using the formula: LC% = (Mass of encapsulated SRF/Mass of initial SRF) × 100 %.

### 4.4 pH-responsive degradation

40 μg of SRF@Fe(III)-COF was added to 1 mL of NaAc solution with indicated pH values (4.5, 5.5, and 7.5), and the mixture was horizontally oscillated at 300 rpm at a temperature of 25 °C. After that, the supernatant was collected to quantify the concentrations of released Fe and SRF using ICP-MS and HPLC [46], respectively.

### 4.5 Measurement of POD-like and GSHox-like activities

SRF@Fe(III)-COF (40 μg/mL, dissolved in NaAc buffer at pH 4.5) was reacted with 3 mM H_2_O_2_ and 1 mM TMB. Following a 60-min incubation period, the absorbance of oxidized TMB was measured to evaluate the POD-like activity. EPR spectroscopy was then employed to verify the production of •OH. In a separate experiment, SRF@Fe(III)-COF (40 μg/mL, dissolved in NaAc buffer at pH 4.5) was incubated with 1 mM DMPO and 3 mM H_2_O_2_ for 10 min, and the DMPO-OH signal was detected using an EPR spectrometer (Bruker EMXplus-6/1, Germany). Additionally, SRF@Fe(III)-COF (40 μg/mL, dissolved in NaAc buffer at pH 4.5) was incubated with 1 mM GSH and 3 mM H_2_O_2_ in the dark for 60 min. Subsequently, the mixture was combined with 1 mM DTNB. After a 5-min incubation, the absorbance of TNB was analyzed to assess the GSHox-like activity [27]. In addition, a 200 μL mixture was incubated with 20 μL GSHtracer (10 mM) for 10 min, and the fluorescence intensities were detected to measure GSH levels using a microplate reader.

### 4.6 Live/dead cell staining

H22 cells were plated at a concentration of 2 × 10^5^ cells per well and subsequently exposed to culture medium supplemented with PBS, Fe(III)-COF (28 μg/mL, according to Fe content), SRF (18 μg/mL, according to SRF LC%), or SRF@Fe(III)-COF (50 μg/mL) for 24 h. After incubation with a calcein-AM/PI staining solution for 10 min, H22 cells were analyzed using a CLSM to obtain the images.

### 4.7 Cell apoptosis

H22 cells were plated at a density of 5 × 10^5^ cells per well and subsequently exposed to PBS, Fe(III)-COF, SRF, or SRF@Fe(III)-COF for 24 h. After incubation with Annexin V-FITC/PI solution, H22 cells were analyzed using flow cytometry.

### 4.8 Sphere formation assay

H22 cells were plated at a density of 600 cells per well in 24-well ultra-low-attachment cell culture plates. These cells were then cultured in a medium supplemented with B27 and insulin for four days [30]. Then, the spheres were treated with PBS, Fe(III)-COF, SRF, or SRF@Fe(III)-COF for 24 h. After the treatment, the spheres were stained using a calcein-AM/PI solution and imaged using CLSM.

### 4.9 Western blot

The cells underwent various treatments and were subsequently lysed in RIPA buffer. Protein samples (40 μg per well) were separated by 12 % SDS-PAGE and transferred to a PVDF membrane using a Bio-Rad gel electrophoresis system. The PVDF membrane was blocked with a 5 % non-fat milk powder solution at room temperature for 1 h, then incubated with an anti-GPX4 antibody at 4 °C overnight. The membrane was further incubated with an HRP-conjugated secondary antibody at room temperature for 2 h. Western blot images were acquired using a Bio-Rad GelDoc XR^+^ system.

### 4.10 Intracellular ROS detection

H22 cells were plated at a density of 1.5 × 10^5^ cells per well in glass-bottom culture dishes and subsequently exposed to PBS, Fe(III)-COF, SRF, or SRF@Fe(III)-COF for 24 h. After incubation with DCFH-DA and MitoPeDPP for 20 min, H22 cells were analyzed using CLSM.

### 4.11 Immunofluorescence

The paraformaldehyde-fixed cells were blocked with normal goat serum containing 0.25 % Triton X-100 for 20 min. Following this, the cells were incubated with indicated antibodies (GPX4, HMGB-1, or CRT) at 4 °C for 16 h. The cells were then incubated with a secondary antibody for 2 h and co-stained with DAPI at room temperature for 10 min. Imaging was performed using CLSM, and the fluorescence intensity was quantified using ImageJ software.

### 4.12 Mouse model

Four-week-old male BALB/c mice were maintained in a specific pathogen-free rodent facility. The animal experiments were approved by the Institutional Animal Care and Use Committee of Wenzhou Institute, CAS (Approval number: WIUCAS23101807). A suspension of H22 cells (1 × 10^6^ per mouse) was injected into the right flank of the mice to establish the H22 tumor-bearing model. On days 7, 10, and 13, the mice were treated with PBS, Fe(III)-COF (56 μg per mouse, according to Fe content), SRF (36 μg per mouse, according to SRF LC%), SRF@Fe(III)-COF (100 μg per mouse), anti-PD-1 antibody (100 μg per mouse), or SRF@Fe(III)-COF + PD-1, respectively. Body weight and tumor volume (tumor volume = (length × width^2^) × 0.5) were measured every three days. On day 19, mouse organs and tumors were collected for further analysis. For the distant tumor model, the primary tumor was injected into the right flank of the mice on day 1. The distant tumor was implanted on the left side on day 4 [40]. Three days later, the mice received combined therapy every 3 days. On day 19, plasma and tumors were collected. The tumor inhibition rate (%) was calculated as (1- W_Treated_/W_PBS_) × 100 %.

### 4.13 Biodistribution

Mice bearing H22 tumors were administered IR820 or IR820@Fe(III)-COF (3 μg per mouse) via intravenous injection. *In vivo* fluorescence imaging was performed and analyzed using the PerkinElmer IVIS Lumina III system. Subsequently, tumor tissues along with major organs were excised for further evaluation of biodistribution through fluorescence imaging.

### 4.14 Histopathological analysis

The paraformaldehyde-fixed tissues were embedded in paraffin for histopathological analysis. The sections were subjected to antigen retrieval and blocked with a 3 % H_2_O_2_ solution for 20 min. The sections were then incubated with normal goat serum containing 0.25 % Triton X-100 for 20 min. Following this, the sections were incubated with indicated antibodies (GPX4, CD8, or Ki67) at 4 °C for 16 h. Then, the sections were incubated with a secondary antibody for 2 h and visualized using the DAB kit. The sections were counterstained with hematoxylin and examined under a microscope.

### 4.15 Flow cytometry analysis

The fresh tumors were dissociated into a single-cell suspension according to a previously published method [47]. Tumor-infiltrating lymphocytes were isolated using Percoll gradient centrifugation. The lymphocytes were then stained with a panel of reagents and antibodies according to the manufacturer's instructions and analyzed using a flow cytometer. The flow cytometric data were analyzed using FlowJo software.

### 4.16 Hemolysis assay

Red blood cells (5 % v/v) were treated with different concentrations of SRF@Fe(III)-COF at 37 °C for 3 h [48]. After centrifugation, the absorbance (540 nm) of the supernatant was measured using a microplate reader.

### 4.17 ELISA

Mouse blood was clotted naturally at 4 °C and centrifuged to obtain the serum. Subsequently, the biochemical indices and cytokine levels in the serum of mice were measured using ELISA kits.

### 4.18 Statistical analysis

Statistical analyses were conducted using GraphPad software. Comparisons between two groups were performed using a two-sided Student's t-test. A p-value of less than or equal to 0.05 was considered to indicate statistical significance.

## CRediT authorship contribution statement

**Binglong Bai:** Writing – original draft, Investigation, Funding acquisition. **Zihao Zheng:** Investigation. **Bingzi Zhu:** Investigation. **Jianlin Wu:** Methodology, Data curation. **Yizhou Xu:** Resources. **Xihao Zhong:** Methodology. **Wenhai Deng:** Software. **Xiang Wang:** Formal analysis. **Shengsheng Zhao:** Data curation. **Tao You:** Resources, Project administration. **Yingpeng Huang:** Validation, Resources. **Weijian Sun:** Supervision, Project administration, Funding acquisition. **Xian Shen:** Writing – review & editing, Supervision, Resources, Funding acquisition. **Xufeng Lu:** Writing – review & editing, Writing – original draft, Supervision, Funding acquisition, Conceptualization.

## Declaration of competing interest

The authors declare that they have no known competing financial interests or personal relationships that could have appeared to influence the work reported in this paper.

## Data Availability

Data will be made available on request.
