# Supplementary material for: An Fe(III)-covalent organic framework (COF)–sorafenib nanoplatform induces chemo-ferroptosis for enhanced hepatocellular carcinoma immunotherapy
Source: Mater Today Bio. 2025 Jul 26;34:102135. doi: 10.1016/j.mtbio.2025.102135 (PMC12337885; doi:10.1016/j.mtbio.2025.102135)
Supplement: Multimedia component 1 [file mmc1.docx]

**Supporting information**

**Chemicals and reagents**

DMTP, TAPB, acetonitrile, acetic acid, p-phenylenediamine, DSPE-PEG_2000_-NHS, FITC, IR820, NaAc, TMB, DMPO, DTNB, GSH, FeCl_3,_ and H₂O₂ were obtained from Shanghai Macklin or Aladdin Biochemical Technology Co., Ltd. Sorafenib tosylate (HY-10201A) was obtained from MedChemExpress (MCE). Annexin V-FITC/PI Apoptosis Detection Kit was purchased from BD Biosciences. FBS, DMEM, RPMI-1640, penicillin‒streptomycin, LIVE/DEAD Fixable Dead Cell Stain Kit, anti-FOXP3 antibody, Ki-67 monoclonal antibody, DAPI, Hoechst 33342, goat anti-rabbit IgG (H+L) cross-adsorbed secondary antibody, Alexa Fluor™ 488, B27, and insulin were obtained from Thermo Fisher Scientific. GAPDH antibody and HRP-linked antibody were purchased from Cell Signaling Technology, Inc. *In vivo* MAb anti-mouse PD-1 antibody was obtained from Bio X Cell. CD8, GXP4, CRT, and HMGB-1 were purchased from Abcam. Cell Counting Kit-8, Cytotoxicity LDH Assay Kit, and Cell Counting Kit-Luminescence (ATP), FerroOrange, MitoPeDPP, Lysotracker, and DCFH-DA were obtained from Dojindo Laboratories. FcX™ PLUS, anti-CD45, anti-CD3, anti-CD4, anti-CD8a antibody, anti-CD44, anti-CD62L, anti-CD49b, anti-B220, anti-CD25 were obtained from BioLegend, Inc. RIPA, protease inhibitor cocktail, and crystal violet, calcein-AM/PI, GSH assay kit, and GSH tracer were purchased from Shanghai Beyotime Biotechnology Co., Ltd. ELISA kits (AST, ALT, BUN, IL-6, TNF-α, IFN-γ) were purchased from Shanghai Jonlnbio Industrial Co., Ltd.

**Characterizations**

The morphology of nanoparticles was analyzed by SEM (ZEISS Sigma 360, Germany) and TEM (JEOL, JEM-F200, Japan). XPS spectra was performed using an XPS instrument (Thermo Fisher Scientific K-Alpha, USA). The hydrodynamic size and zeta potential of nanoparticles were determined using a Zetasizer Nano Analyzer (Malvern, UK). UV‒vis absorption spectra was measured using an UV‒vis spectrophotometer (MAPADA, Shanghai, China). XRD analysis was determined using an X-ray diffractometer (Rigaku Ultima IV, Japan). FTIR spectra was analyzed on an FTIR spectrometer (Thermo Fisher Scientific Nicolet iS20, USA).Brunauer–Emmett–Teller (BET) Measurements were performed on a Micromeritics ASAP 2460.

**Cell culture**

Mouse hepatocellular carcinoma cells (H22), mouse macrophage cells (RAW264.7), and human hepatocellular carcinoma cell lines (HepG2 and Huh-7) were maintained in either Dulbecco's Modified Eagle's Medium (DMEM) or Roswell Park Memorial Institute (RPMI) 1640 medium supplemented with 10% fetal bovine serum (FBS) and 1% penicillin/streptomycin.

**Cellular uptake**

H22 cells were plated at a density of 1.5 × 10^5^ cells per glass culture dish. Subsequently, H22 cells were exposed to either FITC@Fe(III)-COF (10 μg/mL) or FerroOrange (1 μM) for 8 hours. Following a 10-minute incubation with Hoechst and Lysotracker, fluorescence intensity in H22 cells was determined using a CLSM or a flow cytometer.

**LDH and ATP assays**

H22 cells were plated in 96-well plates and subsequently exposed to PBS, Fe(III)-COF, SRF, or SRF@Fe(III)-COF. After a 24-hour incubation, the levels of LDH and ATP were measured using LDH and ATP detection kits, respectively.

**Intracellular Fe content detection**

H22 cells were seeded at a concentration of 1 × 10^6^ cells per well in 6-well plates. After incubation with FITC@Fe(III)-COF (5 μg/mL) for 8 hours, the cells were rinsed three times with PBS. The collected cells were lysed in a 40% HNO₃ solution at 65°C for 8 hours. The lysate was then resuspended in ddH_2_O, and the intracellular Fe content was measured using ICP-MS.

**Cell viability assay**

H22, HepG2, Huh-7 and RAW264.7 cells were plated in 96-well plates and exposed to different concentrations of SRF@Fe(III)-COF for 24 hours. Cell viability was subsequently evaluated using a CCK-8 assay.

**Transwell migration assay**

A transwell system (Corning, REF: 354480) was used to assess the migration of H22. Serum starvation treated-H22 cells (1 × 10^5^) were added to the upper chamber of the transwell insert. The lower chamber was ﬁlled with 600 μL medium containing 20% FBS. After 24 hours of incubation, Hoechst-stained H22 cells that transferred to the lower chamber were directly counted using a CLSM.

**Intracellular GSH detection**

H22 cells were plated at a density of 1 × 10^6^ cells per well and subsequently exposed to PBS, Fe(III)-COF, SRF, or SRF@Fe(III)-COF. After an incubation period of 24 hours, GSH levels were assessed using a GSH assay kit.

**Chemotaxis of RAW264.7 macrophages**

H22 cells were plated at a density of 1 × 10^6^ cells per well in 6-well plates and subsequently treated with PBS, Fe(III)-COF, SRF, or SRF@Fe(III)-COF for 24 hours. Afterward, the medium was centrifuged at 12,000 × g for 20 minutes at 4°C. The supernatant was then transferred to the lower chamber of a transwell insert, and RAW264.7 (1 × 10^5^ per chamber) were seeded into the upper chamber. Following a 24-hour incubation, the migrated RAW264.7 cells were stained with crystal violet.

**Bio-TEM**

The H22 cells were treated with PBS, Fe(III)-COF, SRF, or SRF@Fe(III)-COF for 24 hours, respectively. Then, the cells were fixed with 2.5% glutaraldehyde. Subsequently, the cells were sectioned by a slicer to obtain bio-TEM images.

**Measurement of ROS and GSH in tumor tissues.**

The frozen sections of tumor tissues were stained with DCFH-DA (10 μM) to observe the ROS level and ThiolTracker Violet dye (10 μM) to monitor the GSH level using a CLSM.


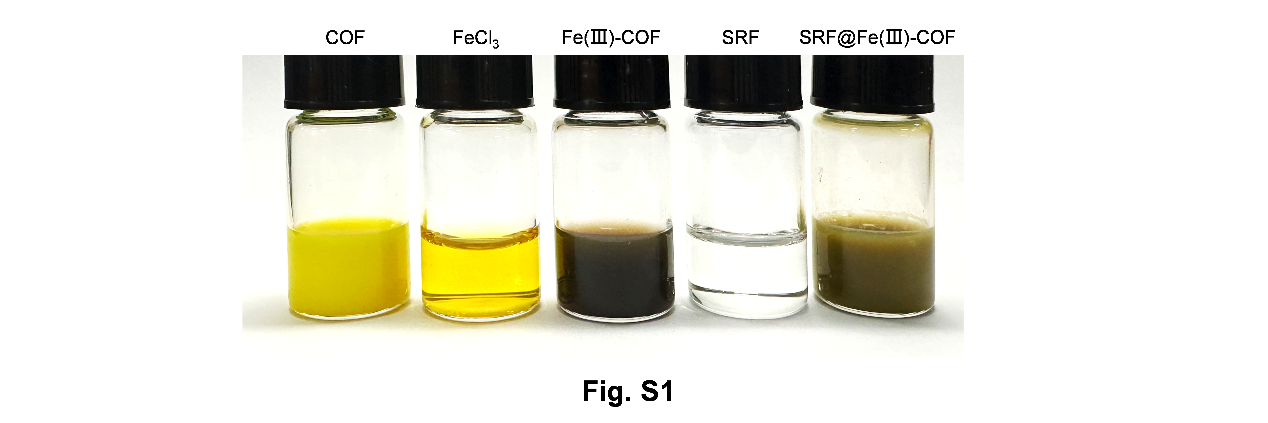


**Fig. S1.** The corresponding images of COF, FeCl_3_, Fe(III)-COF, SRF, and SRF@Fe(III)-COF.


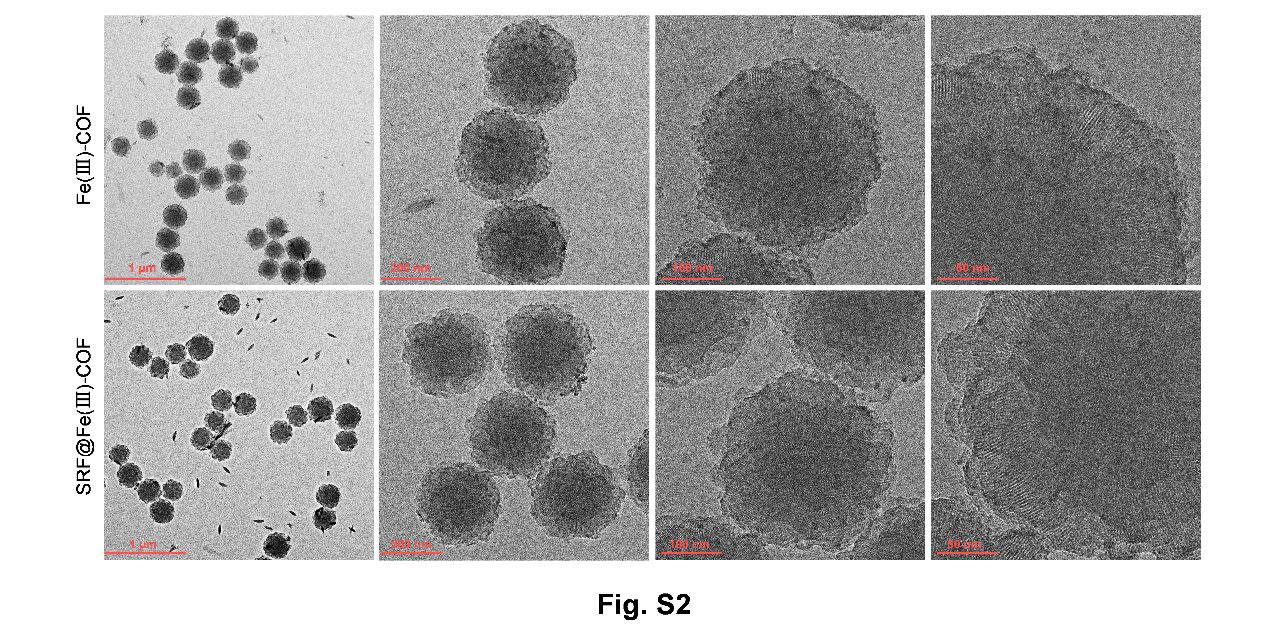


**Fig. S2.** TEM images of the Fe(III)-COF and SRF@Fe(III)-COF.


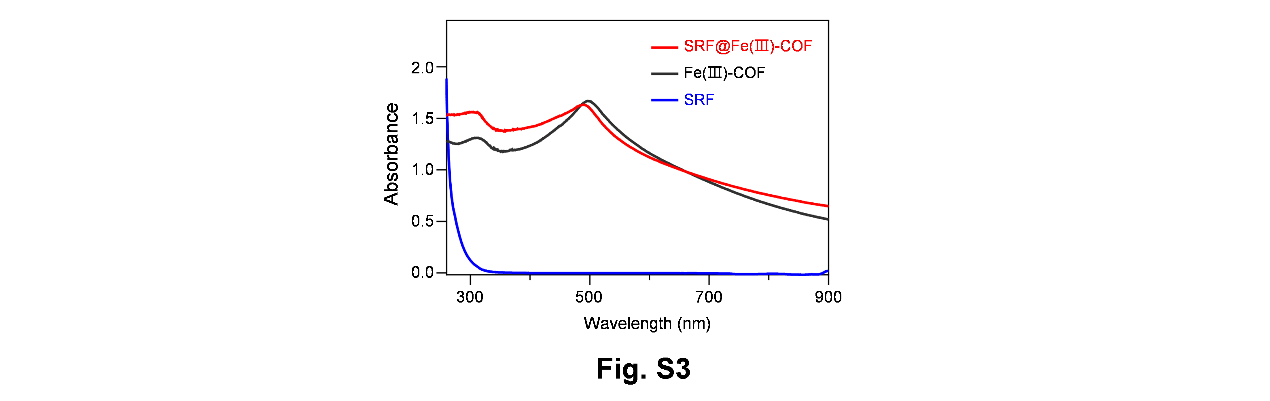


**Fig. S3.** UV‒vis absorption spectra of Fe(III)-COF, SRF, and SRF@Fe(III)-COF.


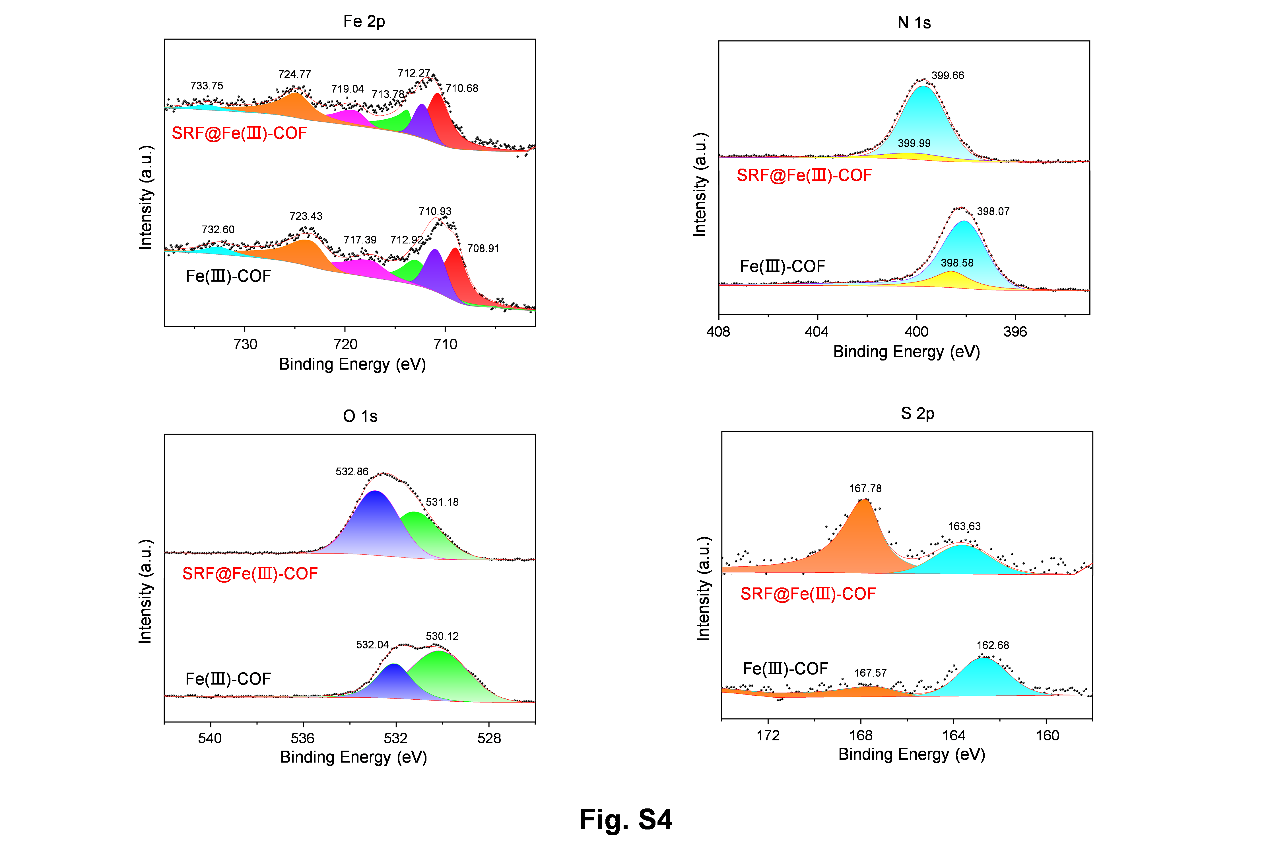


**Fig. S4.** High-resolution XPS spectra of the Fe, N, O, and S orbitals of the Fe(III)-COF and SRF@Fe(III)-COF.


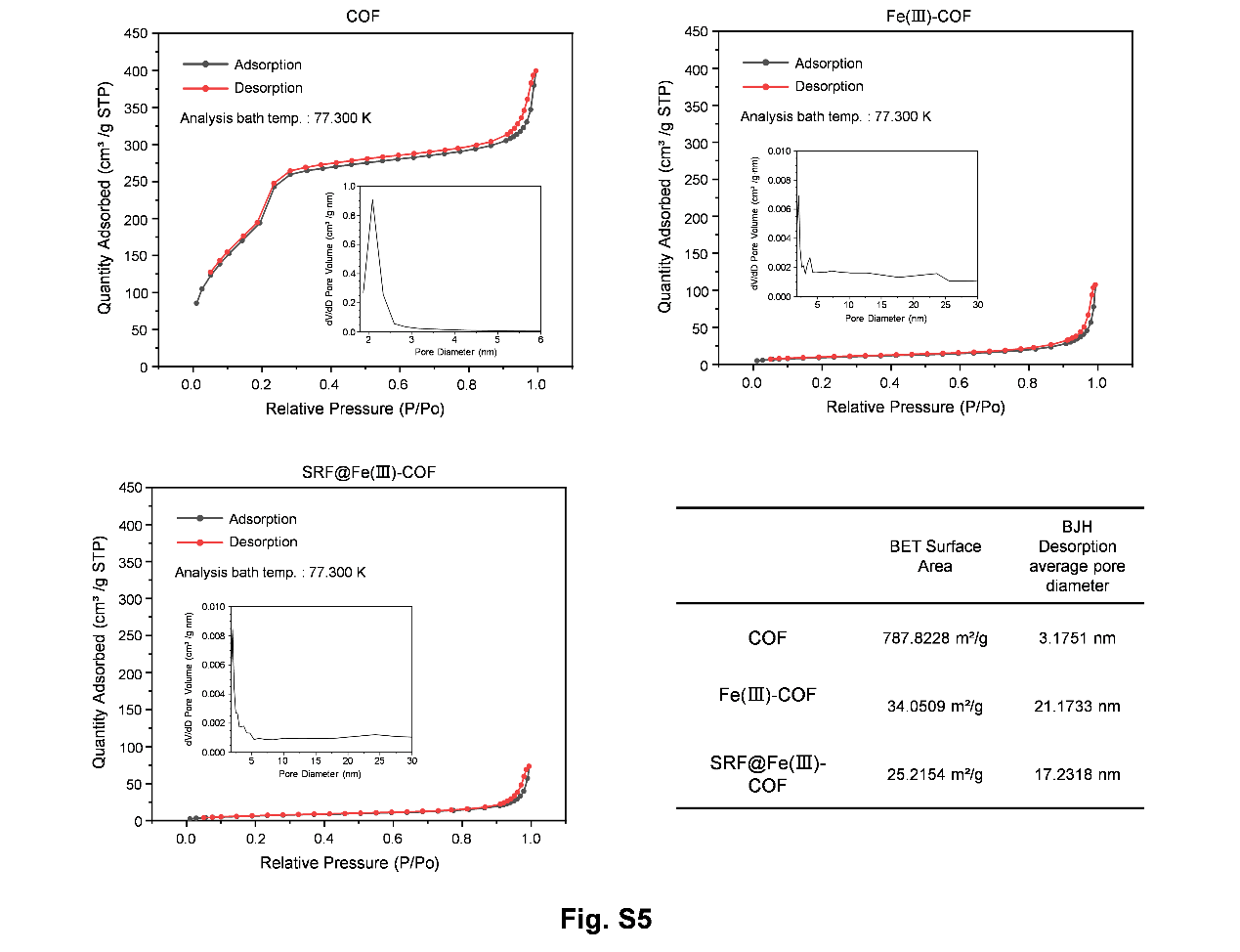


**Fig. S5.** N2 adsorption and desorption isotherms, and pore size distribution plots of COF, Fe(III)-COF, and SRF@Fe(III)-COF.


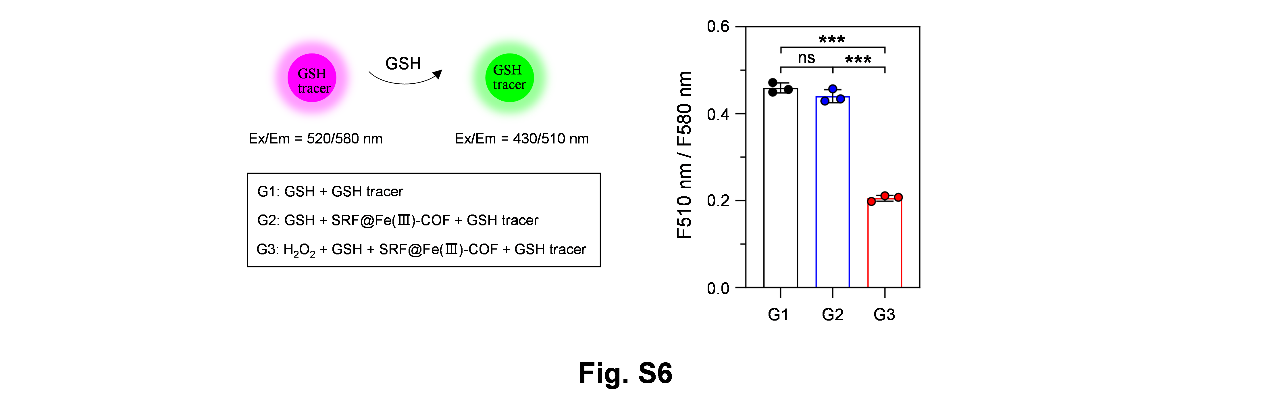


**Fig. S6.** Determination of GSHox-like activity of SRF@Fe(III)-COF using GSH tracer (CAS No: 1479071-34-5) as a substrate. The fluorescence ratio of Em = 510 nm to Em = 580 nm correlated with the GSH depletion (n = 3). Data are presented as mean ± SD; ***p < 0.001; ns, not significant.


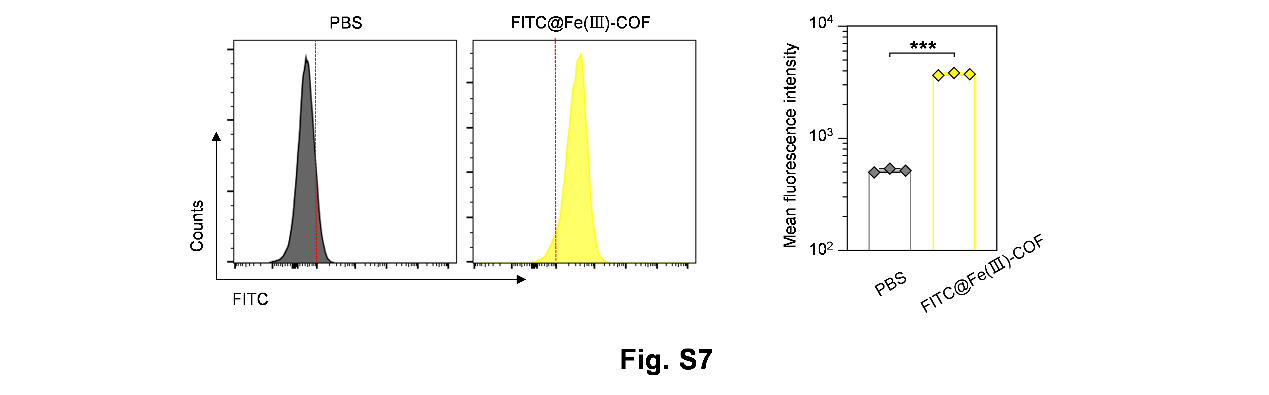


**Fig. S7.** The cellular uptake of FITC@Fe(III)-COF was analyzed by flow cytometry (*n* = 3). Data are presented as mean ± SD; ***p < 0.001.


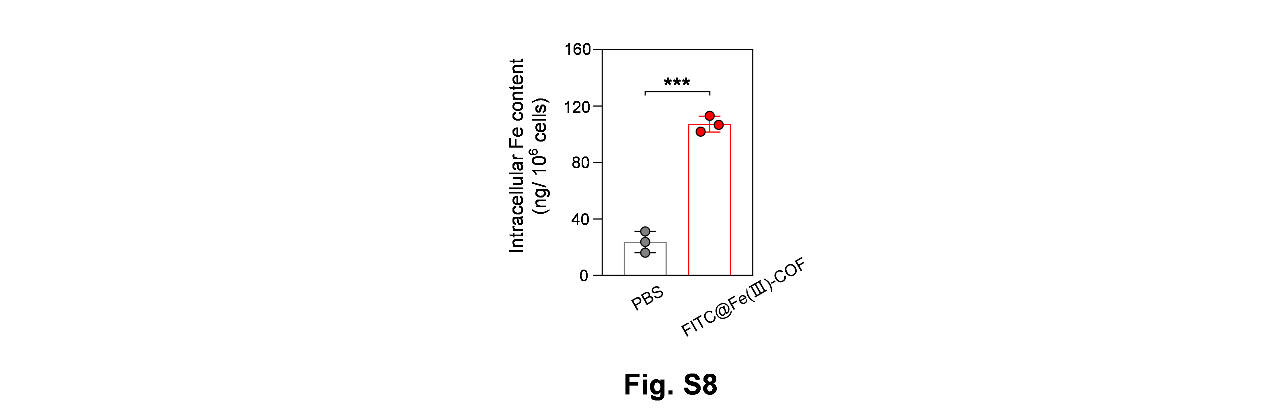


**Fig. S8.** The cellular uptake of FITC@Fe(III)-COF was tracked by ICP-MS (n = 3). Data are presented as mean ± SD; ***p < 0.001.


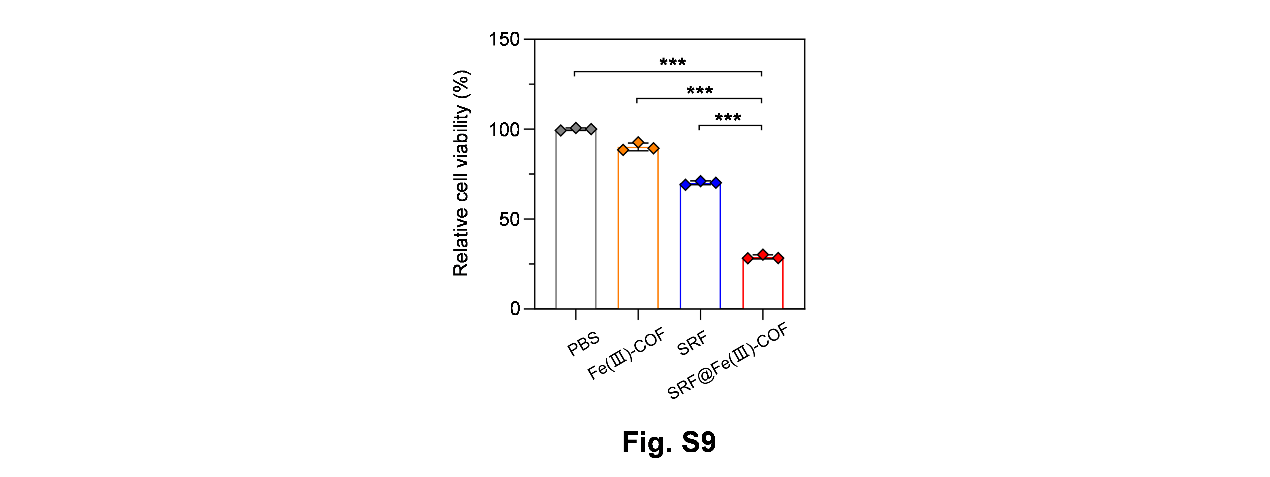


**Fig. S9.** CCK-8 assay in H22 cells treated with Fe(III)-COF, SRF and SRF@Fe(III)-COF (*n* = 3). Data are presented as mean ± SD; ***p < 0.001.


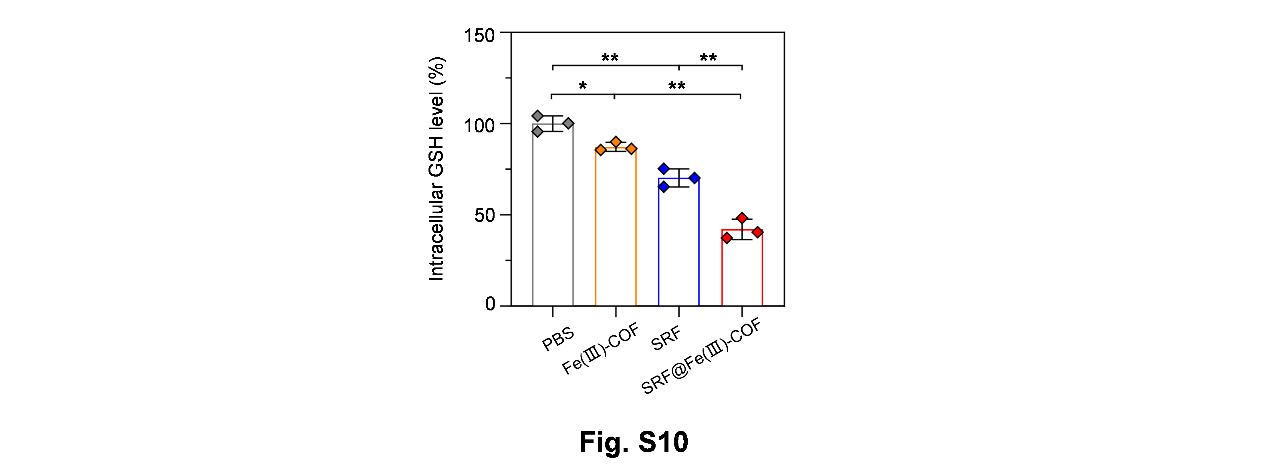


**Fig. S10.** Intracellular GSH levels in H22 cells after the indicated treatments (n = 3). Data are presented as mean ± SD; *p < 0.05; **p < 0.01.


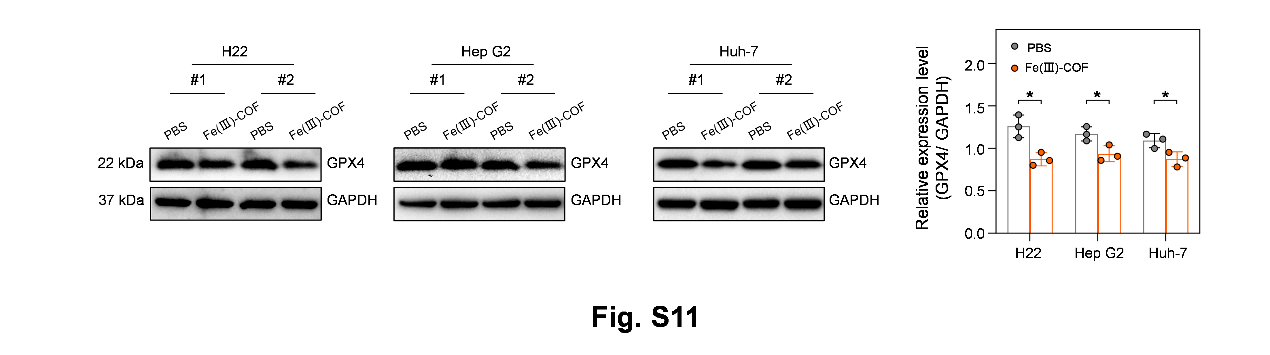


**Fig. S11.** Protein expression of GPX4 in H22, HepG2, and Huh-7 cells after Fe(III)-COF treatment, as evaluated by western blot analysis. GAPDH was used as a loading control. The intensity of the blot was quantified using ImageJ software.


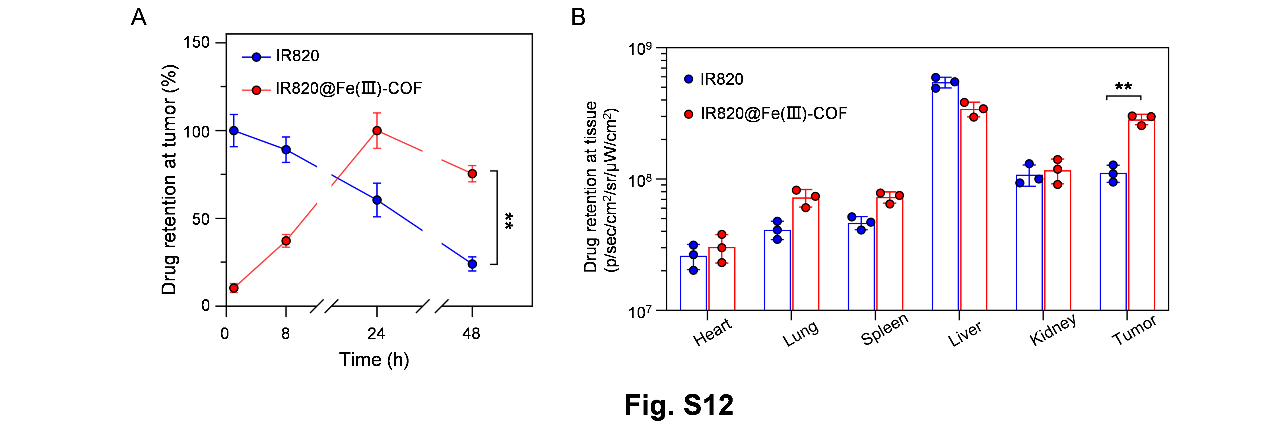


**Fig. S12. *In vivo* Pharmacokinetics.**

A) Quantitative analysis of fluorescence intensity in the tumor area of H22 tumor-bearing mice (n = 3).

B) Quantification of fluorescence intensity in *ex vivo* mouse tumors and major organs collected 48 hours post-injection (n = 3).

Data are presented as mean ± SD; **p < 0.01.


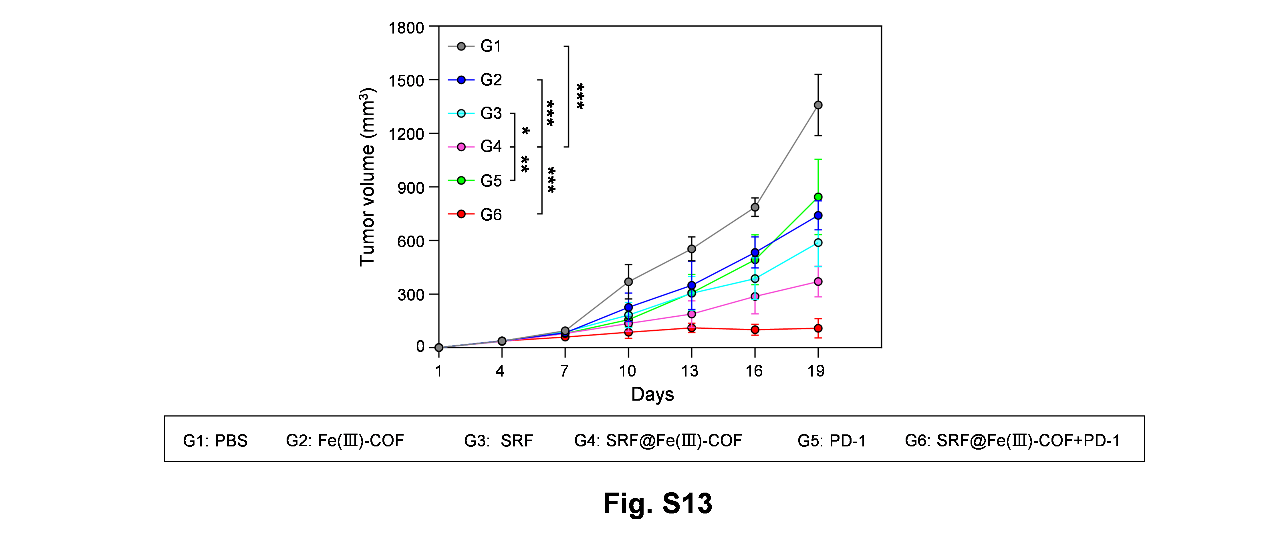


**Fig. S13.** Tumor growth curves of the H22 mouse model following the indicated therapies (*n* = 5). Data are presented as mean ± SD; *p < 0.05; **p < 0.01; ***p < 0.001.


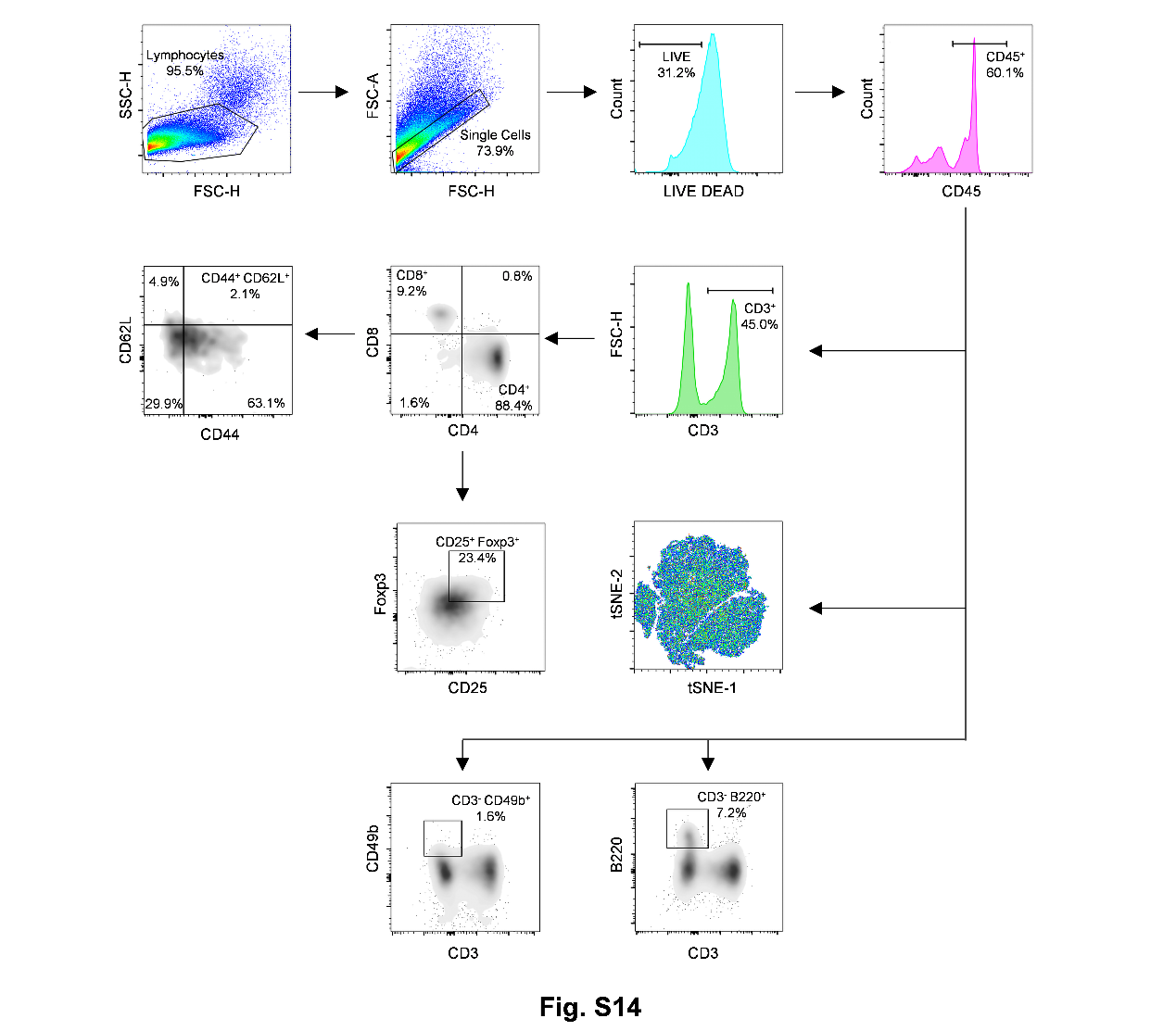


**Fig. S14.** Gating strategy for tumor-infiltrating lymphocytes in tumor tissues after indicated therapies.


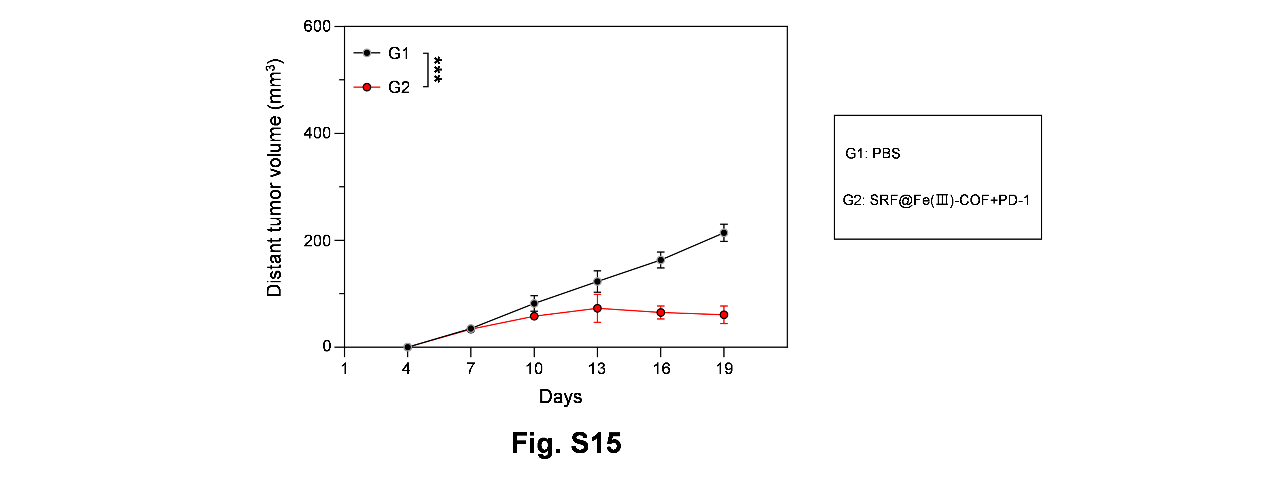


**Fig. S15.** Growth curves of distant tumors in the H22 mouse model following the indicated therapies (*n* = 5). Data are presented as mean ± SD; ***p < 0.001.


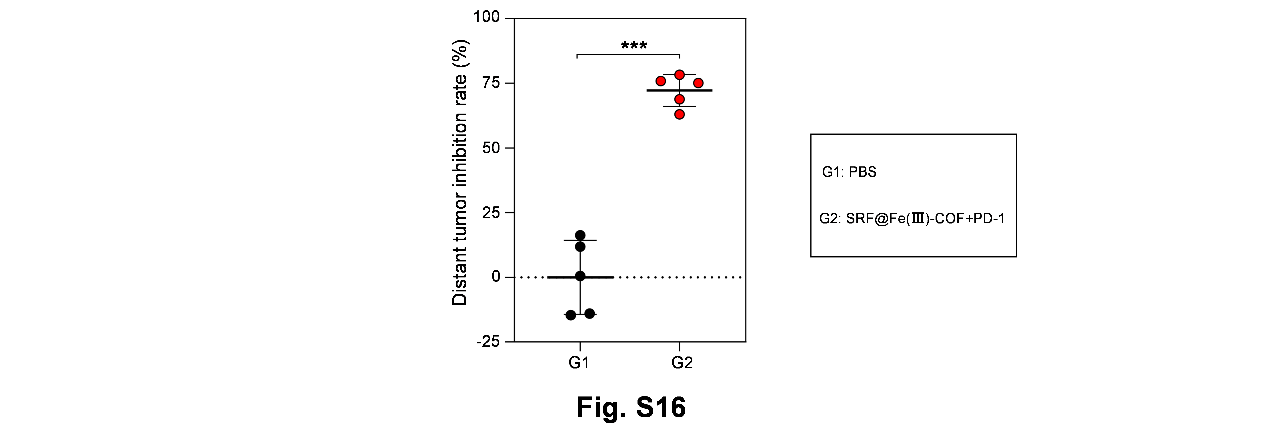


**Fig. S16.** Distant tumor inhibition rates (n = 5). Data are presented as mean ± SD; ***p < 0.001.


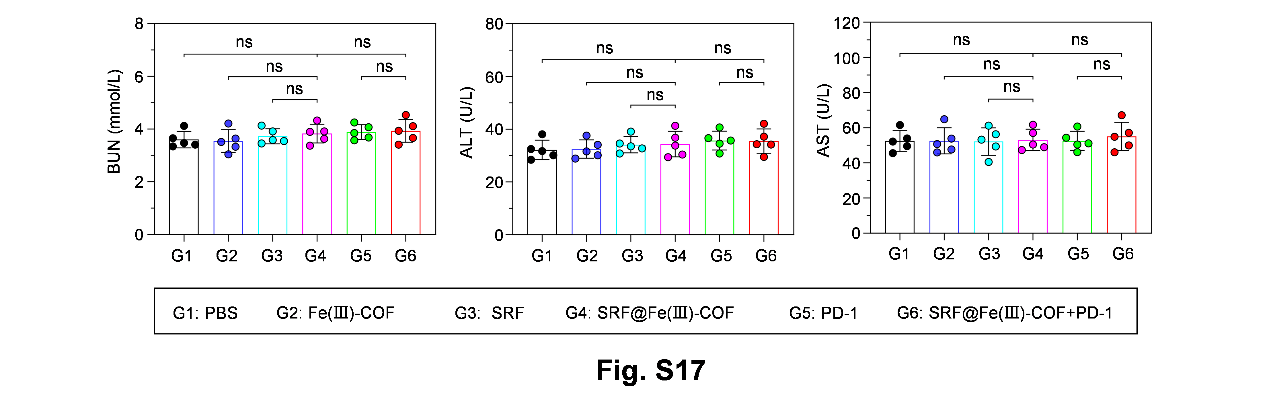


**Fig. S17.** Serum biochemical analysis of kidney function (BUN) and liver function (AST and ALT) after indicated therapies (n = 5). Data are presented as mean ± SD; ns, not significant.


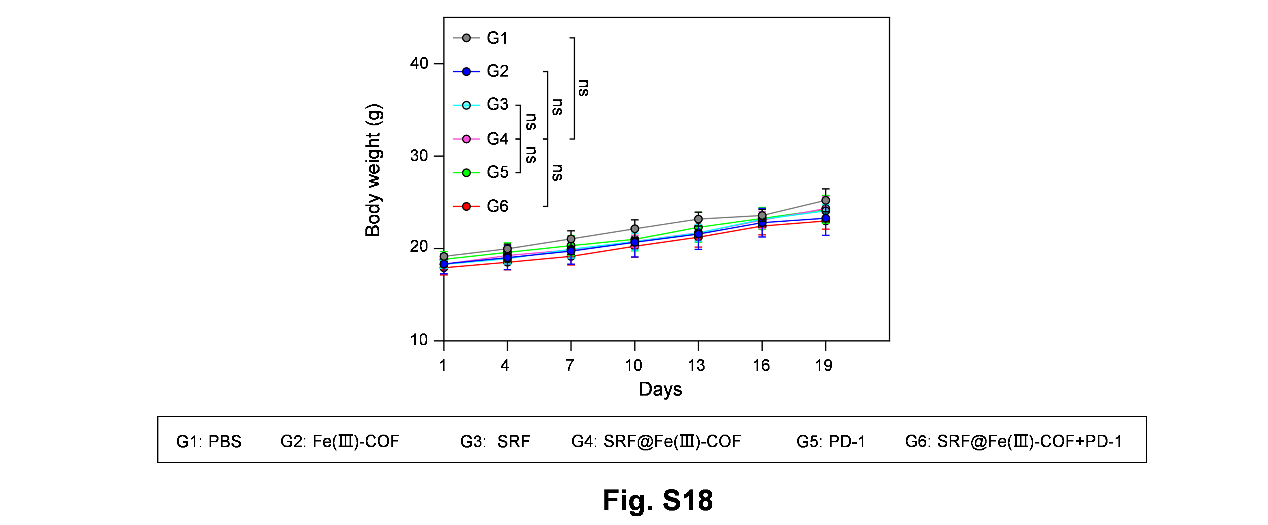


**Fig. S18.** Body weight of H22 tumor-bearing mice during the observation period (n = 5). Data are presented as mean ± SD; ns, not significant.


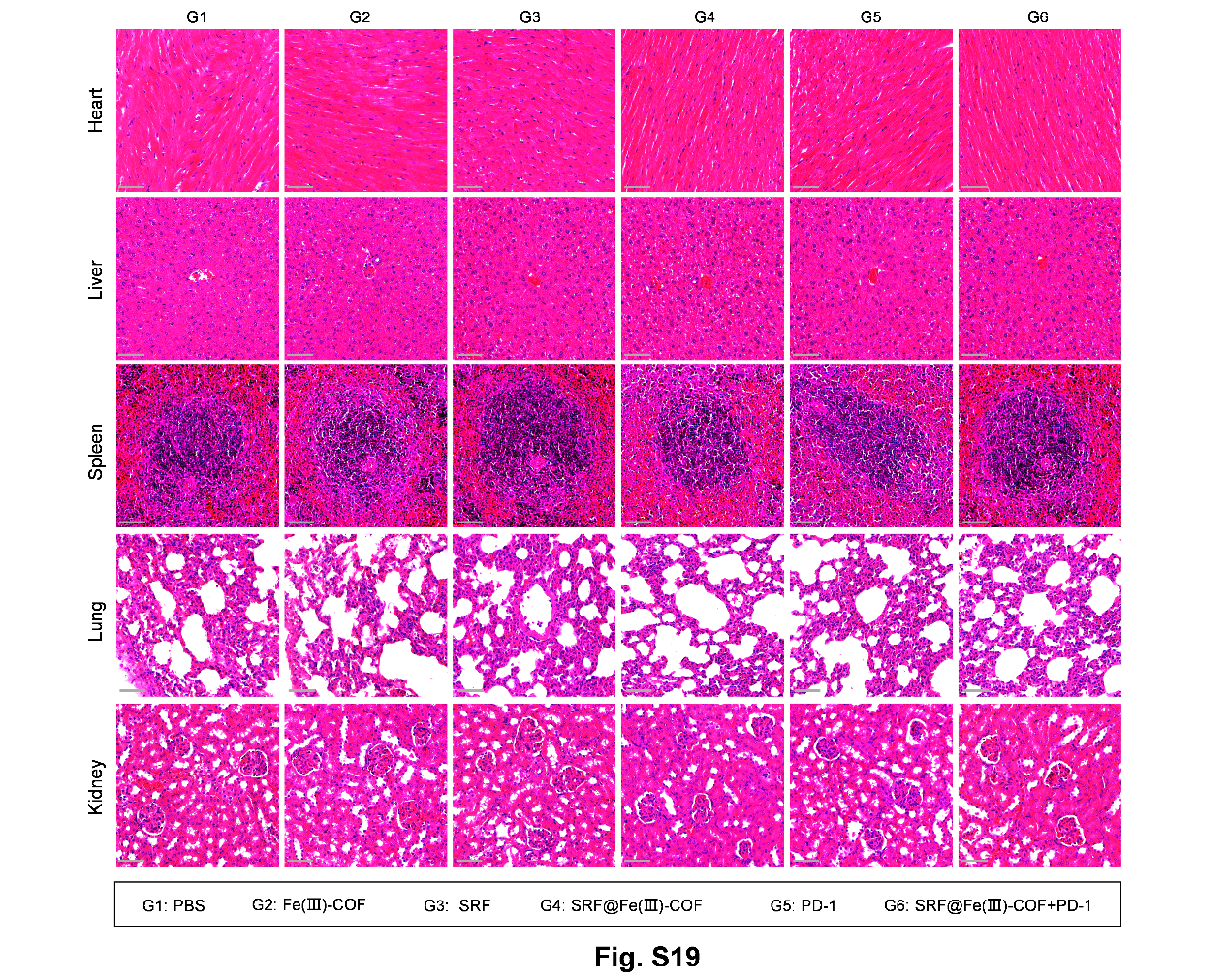


**Fig. S19.** H&E images of major organs collected from the H22 mouse model after indicated therapies. Scale bar: 50 μm.


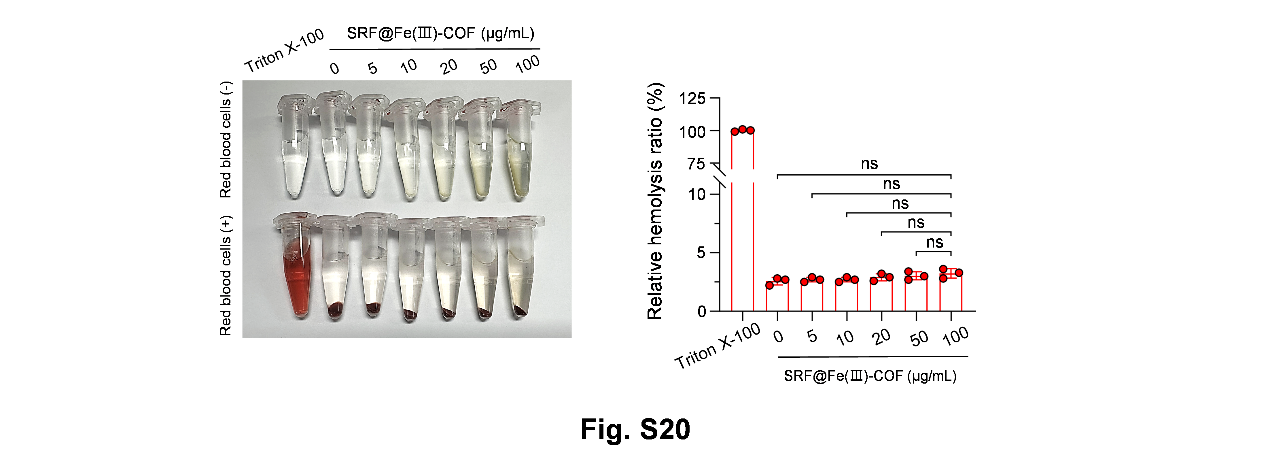


**Fig. S20.** Hemolysis assay of SRF@Fe(III)-COF at different concentrations (n = 3). 0.2% Triton X-100 was used as the positive control. Data are presented as mean ± SD; ns, not significant.


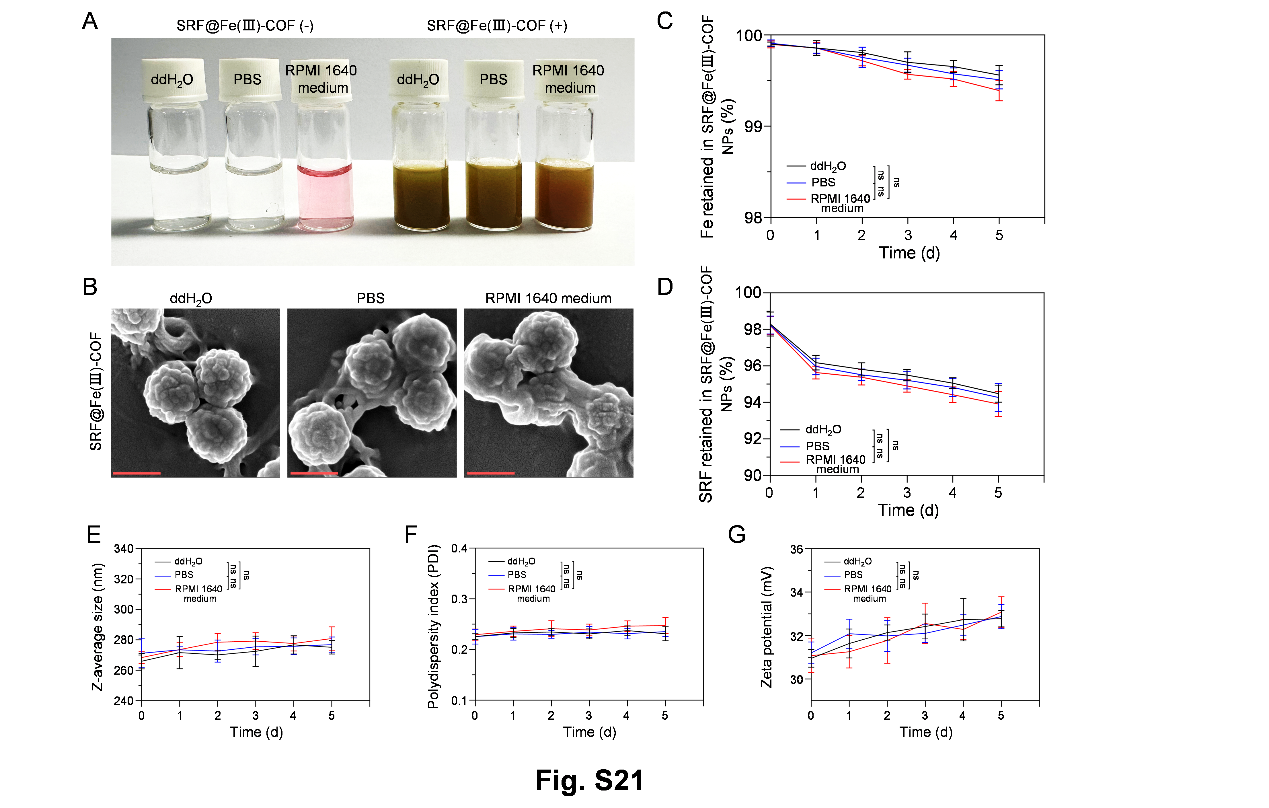


**Fig. S21.** Colloidal stability of SRF@Fe(III)-COF.

A, B) Digital photographs and SEM of SRF@Fe(III)-COF in ultrapure water (ddH_2_O), PBS, and RPMI-1640 medium after incubation for 5 days. Scale bars: 200 nm.

C, D) The time-dependent measurements of released Fe and SRF from SRF@Fe(III)-COF when incubated in ddH_2_O, PBS, and RPMI-1640 medium (n = 3).

E, F) Time-dependent DLS and PDI measurements (n = 3).

G) Time-dependent zeta potential measurements (n = 3). Data are shown as mean ± SD; ns, not significant.


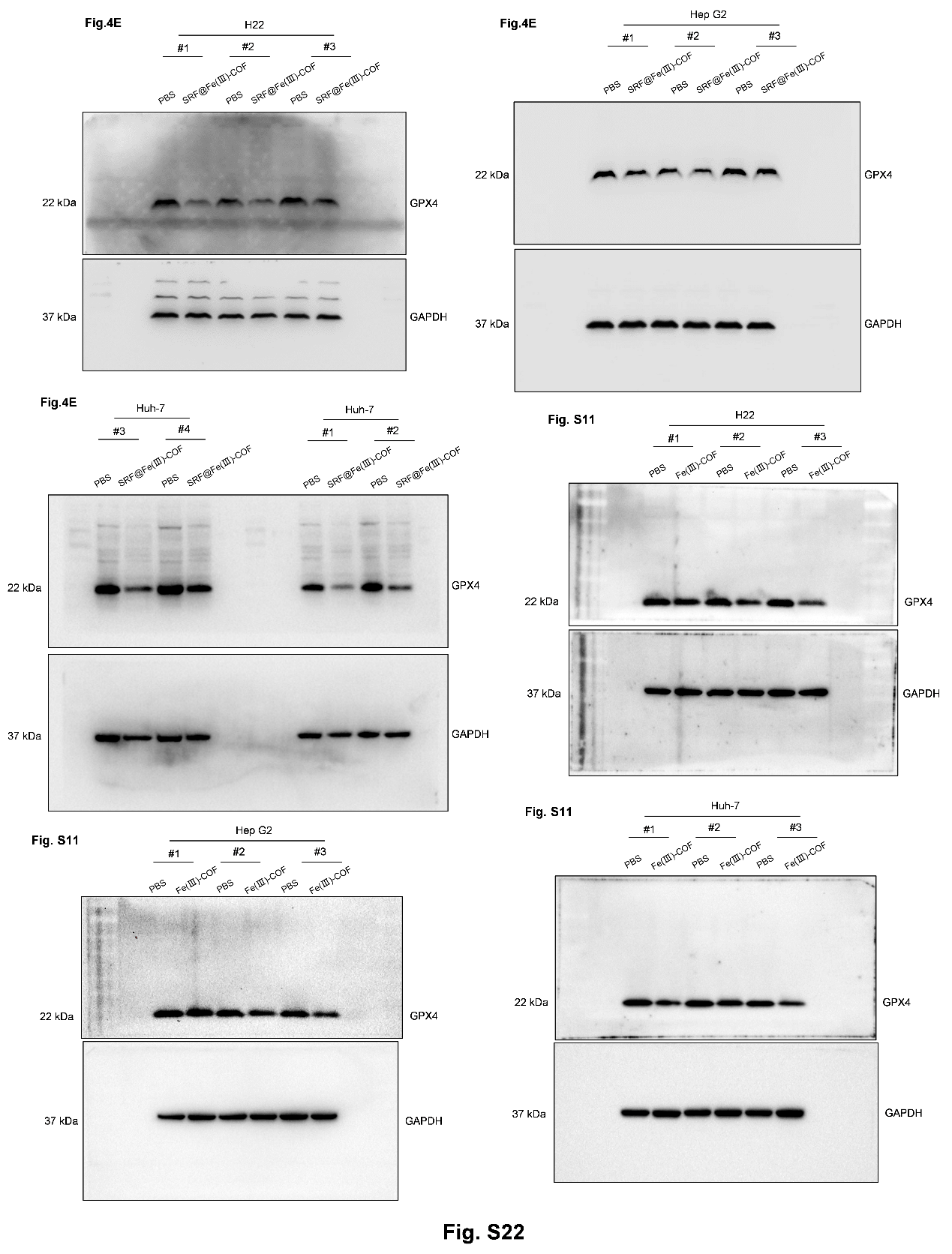


**Fig. S22.** Unprocessed western blots.
